# The molecular basis of μ-opioid receptor signaling plasticity

**DOI:** 10.1038/s41422-025-01191-8

**Published:** 2025-11-07

**Authors:** Huibing Zhang, Xueting Wang, Kun Xi, Qingya Shen, Jianheng Xue, Yanqing Zhu, Shao-Kun Zang, Tianqiang Yu, Dan-Dan Shen, Jia Guo, Li-Nan Chen, Su-Yu Ji, Jiao Qin, Yingjun Dong, Mingming Zhao, Ming Yang, Haijing Wu, Guoli Yang, Yan Zhang

**Affiliations:** 1 Department of Pathology of Sir Run Run Shaw Hospital, Department of Pharmacology, MOE Frontier Science Center for Brain Research and Brain-Machine Integration, and Liangzhu Laboratory, Zhejiang University School of Medicine, Hangzhou, Zhejiang China; 2 Department of Endocrinology and Metabolism, Sir Run Run Shaw Hospital, Zhejiang University School of Medicine, Hangzhou, Zhejiang China; 3 Stomatology Hospital, School of Stomatology, Zhejiang University School of Medicine, Zhejiang Provincial Clinical Research Center for Oral Diseases, Zhejiang Key Laboratory of Oral Biomedical, Hangzhou, Zhejiang China; 4 Department of Dermatology, Hunan Key Laboratory of Medical Epigenomics, The Second Xiangya Hospital, Central South University, Changsha, Hunan China

**Keywords:** Cryoelectron microscopy, Molecular biology

## Abstract

Activation of the μ-opioid receptor (μOR) alleviates pain but also elicits adverse effects through diverse G proteins and β-arrestins. The structural details of μOR complexes with G_z_ and β-arrestins have not been determined, impeding a comprehensive understanding of μOR signaling plasticity. Here, we present the cryo-EM structures of the μOR–G_z_ and μOR–βarr1 complexes, revealing selective conformational preferences of μOR when engaged with specific downstream signaling transducers. Integrated receptor pharmacology, including high-resolution structural analysis, cell signaling assays, and molecular dynamics simulations, demonstrated that transmembrane helix 1 (TM1) acts as an allosteric regulator of μOR signaling bias through differential stabilization of the G_i_-, G_z_-, and βarr1-bound states. Mechanistically, outward TM1 displacement confers structural flexibility that promotes G protein recruitment, whereas inward TM1 retraction facilitates βarr1 recruitment by stabilizing the intracellular binding pocket through coordinated interactions with TM2, TM7, and helix8. Structural comparisons between the G_i_-, G_z_-, and βarr1-bound complexes identified a TM1-fusion pocket with significant implications for downstream signaling regulation. Overall, we demonstrate that the conformational and thermodynamic heterogeneity of TM1 allosterically drives the downstream signaling specificity and plasticity of μOR, thereby expanding the understanding of μOR signal transduction mechanisms and providing new avenues for the rational design of analgesics.

## Introduction

G protein-coupled receptors (GPCRs) possess a transmembrane domain (TMD) that is crucial for signal transduction across the cell membrane. The TMD is composed of a bundle of seven transmembrane α-helices, connected by three extracellular loops (ECLs) and three intracellular loops (ICLs).^1^ The largest phylogenetic class of GPCRs, class A, is characterized by its TMD, which is usually accompanied by short amino termini and carboxyl termini of varying lengths.^2,3^ GPCRs are highly flexible and tend to adopt different conformational states upon interacting with various ligands or downstream signaling effectors, with each state associated with specific physiological responses.^3–5^ The dynamics of an activated GPCR reflect both intrinsic structural changes and extrinsic disturbances resulting from ligand binding or transducer coupling.^1^ Upon transitioning from the inactive to the active state, transmembrane helix 6 (TM6) of GPCRs undergoes a significant rotation and outward displacement, the latter often exceeding 10 Å relative to the central axis of the helical bundle. This movement is accompanied by both rotation and subtle inward displacement of TM5 and TM7.^6,7^ Although the conformational alterations in TMs 5–7 have been well characterized by GPCR structural studies, changes in other transmembrane segments — particularly TM1, which is located at the periphery of the TM core — have received limited attention. Our observations from numerous cryo-electron microscopy (cryo-EM) structures indicate greater flexibility in TM1 than in other TMs. The potential regulatory effects of TM1’s flexibility on the adjacent TM2 and TM7–helix8 interface, as well as its possible involvement in modulating the ligand binding and downstream signaling of GPCRs, remain to be elucidated. Addressing these questions would enhance our understanding of GPCR signal transduction regulation. Incorporating these insights into the drug development process could facilitate the design of innovative conformation-specific pharmaceuticals with improved therapeutic characteristics. Indeed, analysis of a GPCR’s conformational selectivity when bound to different downstream signaling proteins not only enhances our understanding of GPCR dynamics but also facilitates biased ligand design targeting key GPCRs.^8^ In this study, we focused on the μ-opioid receptor (μOR) — a pivotal GPCR that engages multiple downstream signaling pathways and for which the development of signaling-biased ligands with diminished side effects is required — to investigate the precise relationship between TM helix dynamics and downstream signaling regulation.

**Fig. 1:**
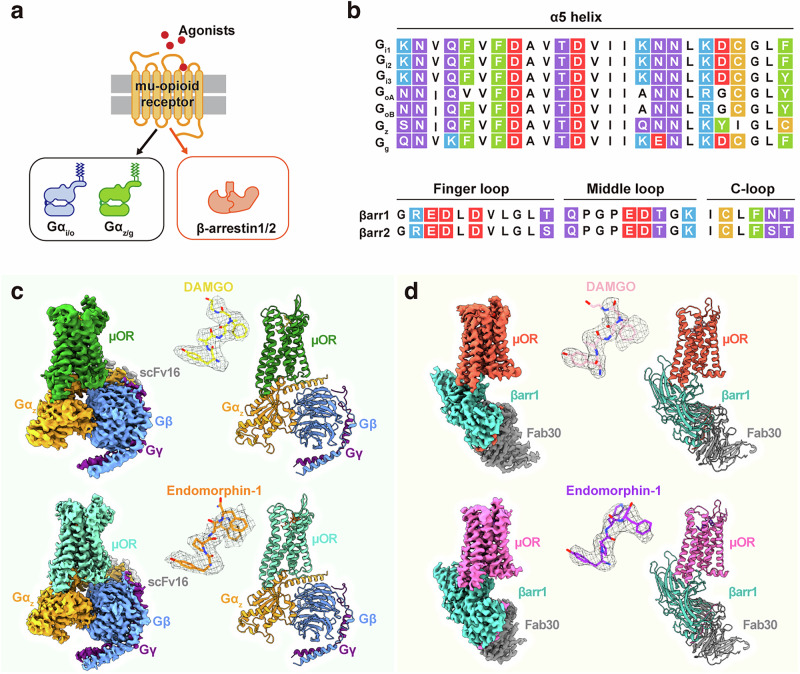
Cryo-EM structures of the μOR–G_z_ and μOR–βarr1 complexes. **a** Schematic diagram of the downstream signal transduction of μOR. **b** Sequence alignment of the Gα_i/o_ family α5 helices and the βarr1/2 finger loop, middle loop, and C-loop. C with an orange background indicates cysteine; F and Y with a green background indicate aromatic amino acids (phenylalanine and tyrosine); S, Q, N, and T with a purple background indicate neutral polar amino acids (serine, glutamine, asparagine, and threonine); K and R with a blue background indicate basic amino acids (lysine and arginine); D and E with a red background indicate acidic amino acids (aspartic acid and glutamic acid); and V, I, L, A, G, and P with a white background indicate nonpolar amino acids (valine, isoleucine, leucine, alanine, glycine, and proline). **c** Cryo-EM density maps and cartoon representations of the μOR–G_z_–scFv16 complexes colored by subunit (gold, DAMGO; forest green, DAMGO-bound μOR; dark orange, endomorphin-1; medium aquamarine, endomorphin-1-bound μOR; orange, Gα_z_; cornflower blue, Gβ; purple, Gγ). **d** Cryo-EM density maps and cartoon representations of the μOR–βarr1–Fab30 complexes colored by subunit (light pink, DAMGO; tomato, DAMGO-bound μOR; dark orchid, endomorphin-1; hot pink, endomorphin-1-bound μOR; light sea green, βarr1; gray, Fab30).

The μOR is the primary target of opioid drugs used in the management of pain, acute pulmonary edema, cough, diarrhea, and shivering.^9–12^ However, μOR–opioid binding also causes undesirable side effects such as respiratory depression and addiction.^13^ Consequently, the clinical utility of opioid drugs is often limited by the development of tolerance and dependence. μOR can activate multiple signaling pathways, including those mediated by various subtypes of G_i_ proteins and β-arrestins (βarrs; β-arrestin-1 and β-arrestin-2, also known as arrestin-2 and arrestin-3).^14–16^ Distinct signaling pathways activated by μOR agonists differentially regulate receptor trafficking, desensitization, internalization, and downstream signaling. μOR interacts with all seven members of the G_i/o_ family, including the conventional subtypes (G_i1_, G_i2_, G_i3_, G_oA_, and G_oB_) and the atypical members (G_z_ and G_g_) (Fig. 1a).^17–19^ Although highly conserved, these seven G protein subtypes exhibit structural and functional differences. All members of the G_i/o_ family, except G_z_, are substrates for ADP-ribosylation by pertussis toxin.^20,21^ G_z_, however, possesses unique biochemical properties and regulatory attributes, exhibiting the least sequence similarity to other G_i/o_ family members (Fig. 1b). Consequently, studying the molecular mechanisms of the μOR–G_z_ complex and its downstream signaling pathways is critically important. Extensive efforts have been dedicated to solving the structures of G_i_-coupled μOR.^19,22–27^ However, the molecular basis of μOR–G_z_ and μOR–βarr coupling has remained elusive, impeding both a comprehensive understanding of μOR-mediated signaling through its diverse adaptor proteins and the development of non-addictive, non-respiratory depressant analgesics. Therefore, elucidating the molecular mechanisms by which μOR recognizes and activates G_z_ and βarr is crucial for the development of μOR-targeting therapeutics.

To elucidate the shared activation mechanisms of different ligands across various downstream signaling pathways, we selected two prototypical peptide agonists, DAMGO ([D-Ala^2^, *N*-MePhe^4^, Gly-ol]-enkephalin, i.e., Tyr-D-Ala-Gly-[*N*-MePhe]-Gly-ol) and endomorphin-1 (Tyr-Pro-Trp-Phe-NH_2_), for cryo-EM structural determination of the μOR–G_z_ and μOR–βarr1 complexes. Notably, these ligands exhibit distinct μOR binding modes but activate the G_i_, G_z_, and β-arrestin pathways to comparable levels. Furthermore, the structures of their respective μOR–G_i_ complexes have already been elucidated.^23,28^ Here, we report cryo-EM structures of the μOR–G_z_ and μOR–βarr1 complexes at nominal resolutions of 2.8 Å (Fig. 1c, d; Supplementary information, Fig. S1 and Table S1). Through comparison with the μOR–G_i_ structures, our study reveals unique conformational changes in μOR that occur upon activation of diverse downstream signaling pathways, particularly the displacement of TM1 relative to other parts of the receptor. DAMGO- and endomorphin-1-coupled μOR–G_z_ and μOR–βarr1 complexes exhibit a conserved conformational shift of TM1 across ligand-specific complexes, which indicates that TM1 is a conserved allosteric regulator of μOR signaling bias. These results, integrated with molecular dynamics (MD) simulations and mutagenesis, establish a correlation between μOR conformational dynamics and signaling outcomes. Furthermore, we identified an allosteric pocket composed of TMs 1, 2, and 7 (designated as the TM1-fusion pocket). In this pocket, ligands with different steric bulk differentially stabilize TM1 conformations, enabling future rational design of biased ligands. Overall, our work establishes a framework for understanding μOR signaling plasticity and implicates TM1 dynamics in the regulation of GPCR signaling, providing mechanistic insights into GPCR signaling diversity and facilitating rational drug discovery.

## Results

### Structures of the μOR–G_z_ and μOR–βarr1 complexes

To further understand μOR–G_z_ coupling and signal transduction, we prepared DAMGO- and endomorphin-1-bound μOR with dominant-negative G_z_ for structural determination (Supplementary information, Fig. S1a, k). The μOR–G_z_ complexes were purified with scFv16 to facilitate cryo-EM studies, which yielded 2.8-Å DAMGO–μOR–G_z_ and endomorphin-1–μOR–G_z_ density maps with clear densities for DAMGO, endomorphin-1, and most side chains of μOR and G_z_ (Fig. 1c; Supplementary information, Figs. S1a–e, k–o, S2a, c and Table S1). The overall architecture of μOR–G_z_ closely resembles that of μOR–G_i_ (root-mean-square deviation (RMSD) of 1.0 Å for Cα). An overlay of G_z_ and G_i_ shows a similar conformation in the α5 helix but a 2.8-Å displacement (measured based on the Cα of Ala7) in the αN helix (Supplementary information, Fig. S3a). The μOR bound to G_z_ exhibits conformational changes similar to those of μOR bound to G_i_, with an outward expansion of TM6 and a downward shift of helix8 (Supplementary information, Fig. S3b). Comparison with the κ-opioid receptor (κOR)–G_z_ complex (PDB: 8dzs)^29^ revealed high structural conservation (Cα RMSD = 0.4 Å), with Gα_z_ adopting a similar α5 helix conformation but showing minor differences in αN helix displacement (Supplementary information, Fig. S3a).

The elastic nature of GPCR–arrestin complexes poses challenges for specimen preparation and structural determination.^30^ We initially assembled the μOR–βarr1 complex using wild-type μOR (μOR^WT^) (Supplementary information, Fig. S1u). However, cryo-EM characterization yielded only a limited subset of viable complex particles. Consequently, 3D reconstruction produced a low-resolution map (6–7 Å), which was insufficient for precise atomic modeling (Supplementary information, Fig. S1v). To optimize the preparation of the μOR–βarr1 complex, we engineered a chimeric μOR (μOR^V2R^) by swapping the C-terminus (P353–P398) of μOR with the C-terminal tail (C342–S371) of the vasopressin 2 receptor (V2R). We then used constitutively active bovine βarr1, truncated at residue 394 with an R169E mutation and the 3 A mutations (I386A, V387A, and F388A); this variant lacks the intrinsic autoinhibition of βarr1, improving the efficiency of complex preparation.^31,32^ Phosphorylation of μOR was achieved by co-expression with G protein-coupled receptor kinase 2 (GRK2) and GRK5. DAMGO and endomorphin-1 were used to stimulate the formation of the μOR–βarr1 complex, which was stabilized with the antibody fragment Fab30^33^ (Supplementary information, Fig. S1f, p). Finally, 2.8-Å maps for the DAMGO-bound μOR–βarr1–Fab30 complex and the endomorphin-1-bound μOR–βarr1–Fab30 complex were obtained by cryo-EM (Fig. 1d; Supplementary information, Figs. S1f–j, p–t, S2b, d and Table S1). These represent the highest-resolution cryo-EM maps of GPCR–arrestin complexes currently available, compared to neurotensin receptor 1 (NTSR1)–βarr1 (PDB: 6pwc, 4.9 Å; PDB: 6up7, 4.2 Å), β_1_-adrenoceptor (β_1_AR)–βarr1 (PDB: 6tko, 3.3 Å), M2 muscarinic receptor (M2R)–βarr1 (PDB: 6u1n, 4.0 Å), V2R–βarr1 (PDB: 7r0c, 4.7 Å), 5-HT_2B_ serotonin receptor (HTR2B)–βarr1 (PDB: 7srs, 3.3 Å), cannabinoid receptor 1 (CB1)–βarr1 (PDB: 8wu1, 3.1 Å; PDB: 8wrz, 3.6 Å), and glucagon receptor (GCGR)–βarr1 (PDB: 8jrv, 3.3 Å; PDB: 8jru, 3.5 Å).^8,30,34–40^ Moreover, the atomic model derived from the high-resolution μOR^V2R^–βarr1 map aligned well with the μOR^WT^–βarr1 cryo-EM map upon rigid-body docking (Supplementary information, Fig. S1w). The high-resolution density maps enabled unambiguous modeling of DAMGO, endomorphin-1, the majority of μOR and βarr1, and associated water molecules (Supplementary information, Fig. S2b, d), providing a structural framework for subsequent conformational analysis.

Notably, both μOR and βarr1 adopt active conformations in the μOR–βarr1 complex (Supplementary information, Fig. S3b, c). Structural comparison between the μOR–βarr1 complex and inactive μOR^26^ reveals that the 7TM bundle of the βarr1-coupled receptor adopts an active-state configuration, characterized by a pronounced outward displacement of TM6 (Supplementary information, Fig. S3b). Compared to the G protein-bound μOR complexes, the density of ICL3 is absent in the μOR–βarr1 complex (Supplementary information, Fig. S3b). The activation of βarr1 is marked by a 15° rotation of the C-domain relative to the N-domain, along with active conformational changes in the central crest loops, as demonstrated by comparison with its inactive form^41^ (Supplementary information, Fig. S3c). In the structure of the µOR–βarr1 complex, βarr1 engages the receptor core in a manner similar to the β_1_AR–, CB1–, and M2R–βarr1 complexes, rather than the V2R–, NTSR1–, or HTR2B–βarr1 complexes (Supplementary information, Fig. S3d).

### TM1 and helix8 dynamics in various adaptor couplings of µOR

**Fig. 2:**
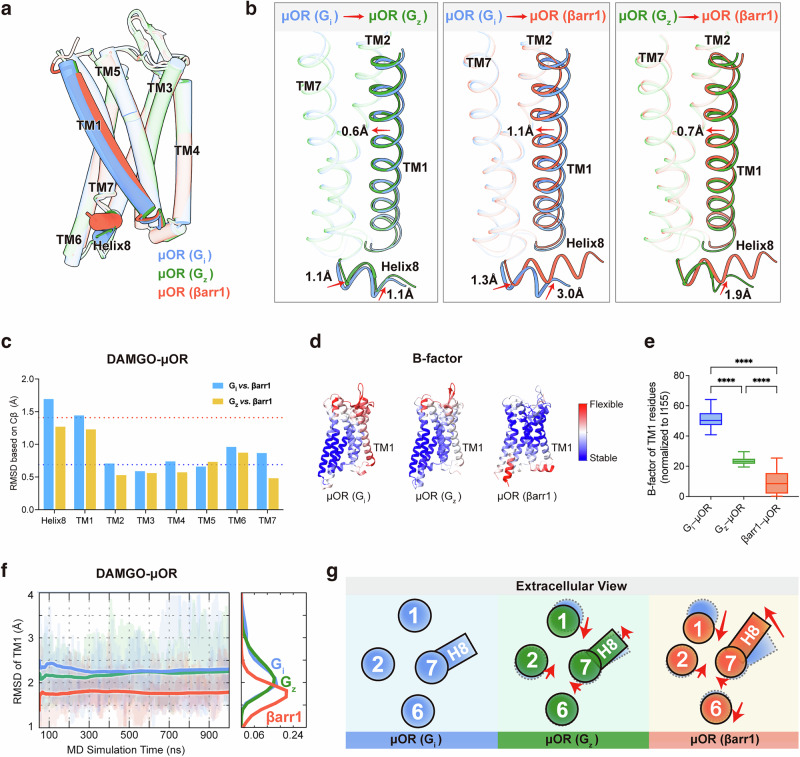
TM1 and helix8 dynamics in various adaptor couplings of µOR. **a**, **b** Superposition of βarr1-coupled μOR (tomato), G_z_-coupled μOR (forest green), and G_i_-coupled μOR (cornflower blue; PDB: 8efq) activated by DAMGO (**a**), highlighting the changes in TM1 and helix8 in pairwise superpositions (**b**). TM1 and helix8 movements are indicated by red arrows. **c** RMSD comparison of TMs 1–7 and helix8 of μOR (comparing G_i_ vs βarr1 and G_z_ vs βarr1-coupled states) in the DAMGO-activated state. The red dashed line represents the average RMSD of TM1 and helix8, and the blue dashed line represents the average RMSD of TMs 2–7. **d** G_i_-, G_z_-, and βarr1-coupled μOR activated by DAMGO, colored by B-factors. **e** Statistics of the normalized B-factor values of TM1 residues (T^1.34^–I^1.57^) in DAMGO-activated μOR coupled with G_i_, G_z_, and βarr1. The B-factor of I^3.40^ in each complex was set to 0 Å^2^ as a reference point. **f** Ensemble average of MD simulation results for TM1 dynamics in βarr1-coupled μOR (tomato), G_z_-coupled μOR (forest green), and G_i_-coupled μOR (cornflower blue; PDB: 8efq) complexes activated by DAMGO. **g** Schematic representation of inter-helical movements with arrows indicating the direction of movement.

Comparative structural analyses of μOR coupled to G_z_, βarr1, and G_i_ reveal how μOR conformational changes precisely regulate coupling to downstream transducers (Fig. 2a; Supplementary information, Fig. S3b). The most pronounced conformational differences in μOR across the three signaling complexes are found in TM1 and helix8 (Fig. 2). In the G_z_-coupled µOR activated by DAMGO, notable alterations compared to the G_i_-bound µOR include a 1.1-Å upward displacement of helix8 (measured at the Cα of R^8.52^; superscript denotes Ballesteros–Weinstein numbering^42^), a 1.1-Å outward movement of the TM7–helix8 hinge in the cytoplasm (measured at the Cα of E^8.48^), and a 0.6-Å inward displacement of the entire TM1 segment (measured as the average displacement among the Cα of I^1.33^–Y^1.60^) (Fig. 2b, left panel). For the βarr1-coupled µOR, the most evident changes compared to the G_i_-bound µOR are a 3.0-Å upward displacement of helix8, a 1.3-Å outward movement of the TM7–helix8 hinge in the cytoplasm, and a 1.1-Å net inward displacement of the entire TM1 segment; the TM1 inward displacement is 0.7 Å relative to the µOR bound to G_z_ (Fig. 2b, middle and right panels). These three changes — upward displacement of helix8, outward movement of the TM7–helix8 hinge, and inward displacement of TM1 — are also observed in the endomorphin-1–µOR–βarr1 and –G_z_ complexes relative to the endomorphin-1–µOR–G_i_ complex (Supplementary information, Fig. S4a). The insertion of the fourth residue phenylalanine (Phe4) of endomorphin-1 into the pocket formed by TMs 1, 2, and 7 results in a 0.4-Å inward movement of TM1 in the endomorphin-1–µOR–βarr1 complex compared to the endomorphin-1–µOR–G_i_ complex, which is slightly less significant than that observed between the DAMGO–µOR–βarr1 and DAMGO–µOR–G_i_ complexes (Supplementary information, Fig. S4a, b). Overall, the trend in TM1 movement in endomorphin-1-activated signaling complexes aligns with that observed in DAMGO-activated signaling complexes. The RMSD of each segment was calculated across various signaling complexes, showing pronounced differences in TM1 and helix8 in the G_i/z_- and βarr1-bound µOR complexes (Fig. 2c; Supplementary information, Fig. S4c–e). These observations indicate that the conformational states of µOR — whether it is G_i_-, G_z_-, or βarr1-coupled — are regulated by TM1 and helix8.

To investigate TM1 dynamics, we analyzed B-factors across the three µOR signaling complexes. In the DAMGO–µOR–G_i/z_ complex, TM1 exhibits the highest B-factor among the 7 TMs, indicating enhanced flexibility. In contrast, in the DAMGO–µOR–βarr1 complex, the B-factor of TM1 is comparable to those of other transmembrane segments and lower than in the DAMGO–µOR–G_i/z_ complex, which indicates reduced flexibility (Fig. 2d, e). In the endomorphin-1–µOR–βarr1 complex, the B-factor of TM1 is similar to other transmembrane segments, whereas it is significantly higher in the endomorphin-1–µOR–G_z_ complex (Supplementary information, Fig. S4f). The B-factor of TM1 was not analyzed for the antiparallel endomorphin-1–µOR–G_i_ complex. In previously resolved µOR–G_i_ structures (excluding dimers), TM1 generally displays higher B-factors, suggesting that TM1 has greater flexibility in µOR–G_i_ complexes (Supplementary information, Fig. S4g).

We further conducted MD simulations to evaluate TM1 dynamics in the three µOR signaling complexes (Supplementary information, Fig. S4h). The simulations revealed that the RMSD of TM1 in the G_i_- and G_z_-coupled µOR structures exhibited significant variability, whereas the RMSD of TM1 in the βarr1–µOR complex was significantly smaller than that in the G_i/z_–µOR complexes (Fig. 2f; Supplementary information, Fig. S4i). These findings suggest that TM1 is more stable in the βarr1–µOR signaling complex. In this complex, TM1 is positioned closer to TMs 2 and 7. In addition, helix8 contributes to stabilizing the intracellular portion of TM1, resulting in reduced TM1 conformational flexibility in the βarr1–µOR complex compared with its behavior in the G_i/z_–µOR complexes. Overall, TM1 and helix8 demonstrate pronounced conformational selectivity associated with downstream signaling, which indicates regulatory roles in shaping the structural conformation of µOR and the binding of downstream signaling proteins (Fig. 2g).

**Fig. 3:**
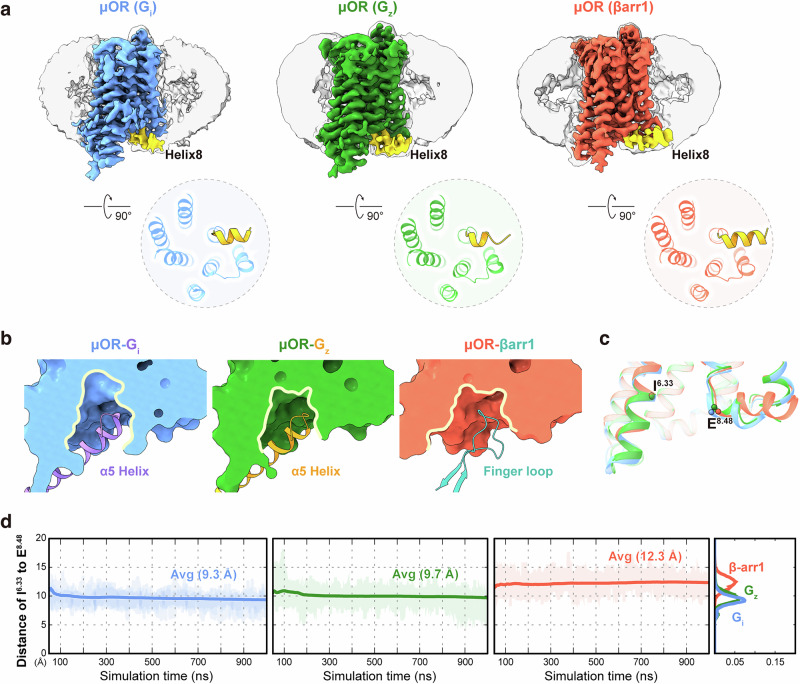
Dynamics of the intracellular binding domain in various adaptor couplings of µOR. **a** Density of helix8 in various cryo-EM density maps and models of µOR coupled with G_i_, G_z_, and βarr1 (cornflower blue, G_i_-coupled μOR; forest green, G_z_-coupled μOR; tomato, βarr1-bound μOR; μOR–G_i_ complex, PDB: 8efq). Helix8 is highlighted in yellow in each case. **b** Comparison of the intracellular halves of the μOR–G_i_, μOR–G_z_, and μOR–βarr1 complexes, shown as surface slice representations to illustrate the formation of the transducer-binding cavity (medium purple, G_i_; orange, G_z_; light sea green, βarr1). **c** Superposed intracellular structures of μOR coupled with G_i_, G_z_, and βarr1, with residues I^6.33^ and E^8.48^ annotated. **d** MD simulations of μOR reveal distinct intracellular pockets, based on the distance distribution and the ensemble average of the distance between I^6.33^ and E^8.48^.

We next investigated the alterations induced by variations in helix8 across diverse signal transduction complexes. The different conformations of helix8 in these signaling complexes demonstrate its critical role in the recruitment of downstream transducers. In the G_i/z_-bound μOR complexes, helix8 undergoes a downward displacement, moving away from the membrane environment, whereas in the μOR–βarr1 complex, it remains within the micelle (Fig. 3a). In the βarr1-bound μOR, helix8 is closer to TM1 and interacts with both TM1 and ICL1, thereby helping to stabilize the inward conformation of TM1 and enlarging the binding pocket for βarr1 (Fig. 3b, c). The α5 helix of G_i/z_ inserts into a similarly sized intracellular pocket of μOR (about 11.3 Å in width, measured by the distance between Cα of I^6.33^ and E^8.48^). The finger loop of βarr1 occupies the same pocket, which shows a similar depth but greater width (12.8 Å) (Fig. 3b, c). To observe the dynamics of the intracellular pocket, we performed MD simulations using the three structural models with the extracellular pocket of μOR held fixed. The results showed that the distance between I^6.33^ and E^8.48^ was greater in the βarr1-stabilized complex (averaging 9.3 Å in G_i_-bound μOR, 9.7 Å in G_z_-bound μOR, and 12.3 Å in βarr1-bound μOR) (Fig. 3d), consistent with our structural observations.

### TM1 regulates µOR’s downstream signaling selectivity

**Fig. 4:**
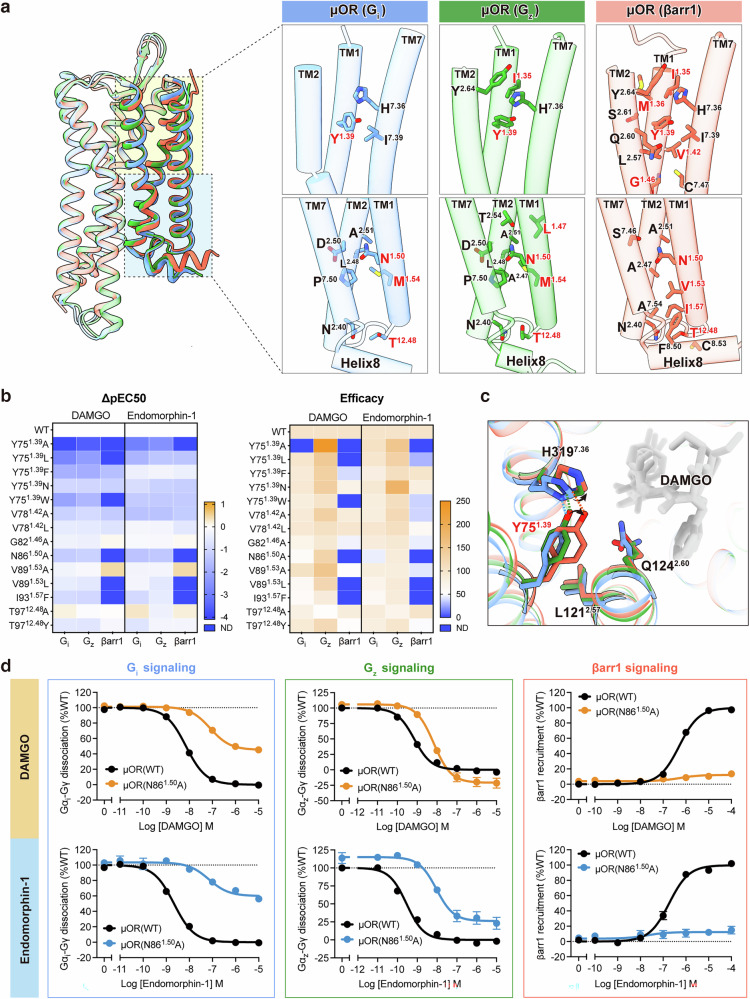
TM1 regulates µOR downstream signaling selectivity. **a** Residues involved in the interaction between the TM1–ICL1 region and the TM2–TM7–helix8 interface of G_i_-, G_z_-, and βarr1-bound μOR activated by DAMGO (cornflower blue, G_i_-coupled μOR; forest green, G_z_-coupled μOR; tomato, βarr1-bound μOR; μOR–G_i_ complex, PDB: 8efq). **b** Mutagenesis analysis of the key residues of TM1 and ICL1 in G_i_-, G_z_-, and βarr1-bound μOR activated by DAMGO and endomorphin-1, measured using a BRET assay. **c** Structural alignment of μOR bound to G_i_, G_z_, and βarr1 showing the position of Y75^1.39^ and its interaction with the TM2/TM7 domain. **d** Mutagenesis analysis of N86^1.50^ in G_i_-, G_z_-, and βarr1-bound μOR activated by DAMGO and endomorphin-1, measured using a BRET assay. Data represent mean ± SEM of n ≥ 3 biological replicates.

Owing to its exterior location and flexibility, TM1 is often overlooked in investigations of GPCR conformational changes. We conducted a detailed analysis of the interactions between TM1 and other components of µOR in each of the three signaling complexes. Our structural analysis revealed that TM1 engages in a range of interactions with the TM2–TM7–helix8 interface in the different μOR signaling complexes. Notably, TM1 forms the most interactions with this interface in the βarr1-coupled µOR complex and the fewest in the G_i_ complex (Fig. 4a). The interactions are similar in the signaling complexes activated by the two different ligands (DAMGO and endomorphin-1) (Supplementary information, Fig. S5a).

To investigate the regulatory role of TM1 in downstream signaling, we mutated residues within TM1 involved in intra-µOR interactions and evaluated their effects on downstream signaling pathways (Fig. 4b). Y75^1.39^, located at the extracellular of TM1 and inserted between TM2 and TM7, forms a hydrogen bond (H-bond) with H319^7.36^ in all three signaling complexes (Fig. 4c; Supplementary information, Figs. S2, S5b). In the DAMGO–µOR–βarr1 complex, Y75^1.39^ is deflected toward TM2 and forms additional hydrophobic interactions with L121^2.57^ and Q124^2.60^, stabilizing key interactions between TM2 (L121^2.57^ and Q124^2.60^) and TM7 (Y326^7.43^) (Fig. 4c). Notably, mutations of Y75^1.39^ to either small- or large-side-chain residues strongly affected βarr1 signaling pathways. Y75^1.39^A and Y75^1.39^L led to a marked reduction in downstream signaling potency, with the greatest effect observed in βarr1 recruitment. In contrast, Y75^1.39^F and Y75^1.39^N only resulted in minor changes in downstream signaling. Furthermore, mutation of Y75^1.39^ to tryptophan nearly abolished βarr1 signaling while maintaining strong signaling through G proteins, particularly G_z_, which indicates that Y75^1.39^ exerts diverse regulatory functions across different signal transduction complexes (Fig. 4b; Supplementary information, Tables S2–S4). Importantly, Y75^1.39^ mutations had a notably weaker effect on μOR activation by endomorphin-1 compared with their influence on DAMGO-induced activation (Fig. 4b; Supplementary information, Fig. S5b and Tables S2–S4). This difference may be due to the insertion of Phe4 of endomorphin-1 into the TM1-fusion pocket formed by TMs 1, 2, and 7, which stabilizes the interaction between TMs 2 and 7 and mitigates the influence of Y75^1.39^.

Other TM1 residues involved in interactions with the TM2–TM7–helix8 interface, when mutated to smaller side chains (V78A, N86A, and V89A) or slightly larger side chains (V78L, G82A, V89L, and I93F), resulted in greater attenuation of βarr1 signaling activity, further highlighting the importance of TM1 interactions in βarr1 recruitment (Fig. 4b, d; Supplementary information, Tables S2–S4). T97^12.48^, located in ICL1, interacts with helix8 of µOR. Alanine substitution of T97^12.48^ reduced G_z_ signaling efficacy by ~50% but had minimal effect on the G_i_ and βarr1 pathways. In contrast, tyrosine substitution (T97^12.48^Y) impaired βarr1 signaling by about 50%, whereas G_i_ and G_z_ activation remained largely unaffected (Fig. 4b; Supplementary information, Tables S2–S4). These results indicate that T97^12.48^ requires optimal steric bulk to maintain the conformation of the G_z_-binding pocket. Substitution with tyrosine introduces excessive volume, which may sterically displace helix8 and compromise its stabilizing role in restraining TM1 dynamics within the µOR–βarr1 complex. Therefore, the T97^12.48^Y mutation had the greatest impact on βarr1 signaling.

Notably, N86^1.50^A substantially impeded βarr1 recruitment while preserving 40%–60% efficacy in G_i_ activation and even enhancing G_z_ signaling (Fig. 4d; Supplementary information, Tables S2–S4). In the μOR complexes, the highly conserved residue N86^1.50^ mediates extensive interactions with TMs 2 and 7; in the DAMGO–μOR–βarr1 complex, these include a direct H-bond with S329^7.46^ and interactions with the backbones of TMs 2 and 7 at the connector region (Fig. 4a). N86^1.50^ coordinates polar interactions that selectively modulate μOR engagement with G_i_, G_z_, and βarr1, indicating a pivotal role of position 1.50 in facilitating biased signal transduction within μOR. The propensity for biased signaling observed after mutations at position 1.50 has also been observed in the apelin receptor.^43^ These findings imply that this residue may be crucial for the regulation of signaling bias across class A GPCRs.

### Propagation of µOR conformational changes

**Fig. 5:**
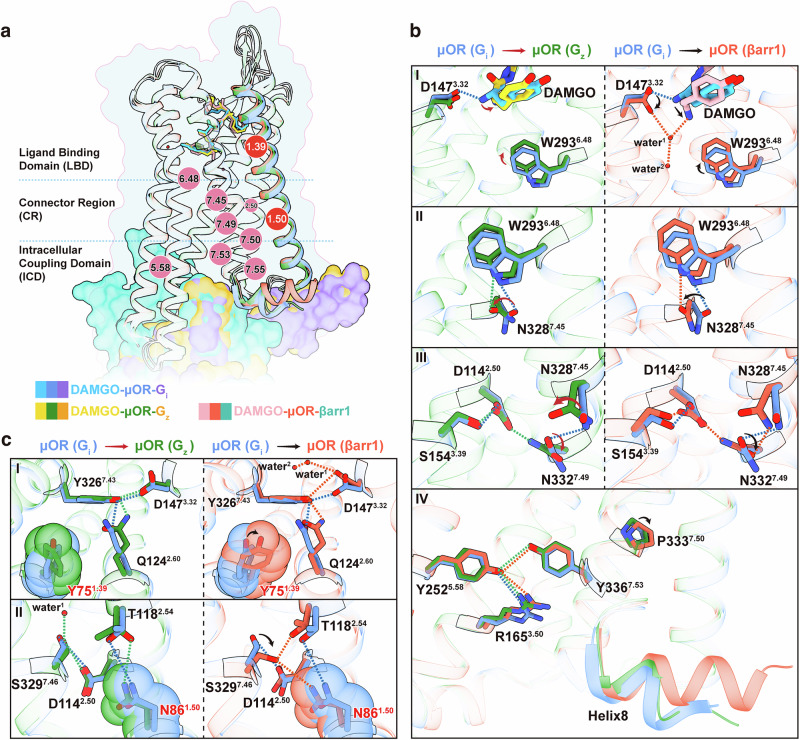
Comparisons of DAMGO-activated μOR in different states. **a** Superposition of βarr1-coupled μOR (tomato), G_z_-coupled μOR (forest green), and G_i_-coupled μOR (cornflower blue; PDB: 8efq) activated by DAMGO. **b**,** c** Close-up views of residues with conformational changes from the G_i_-bound state to the G_z_-coupled state and from the G_i_-bound state to the βarr1-coupled state. Movements of residues and helices from the G_i_-bound state to the G_z_-coupled state are indicated by red arrows, and movements from the G_i_-bound state to the βarr1-coupled state are indicated by black arrows.

Selectivity between G proteins and arrestins has emerged as a central theme in the GPCR field.^3^ Nevertheless, due to the challenges in obtaining high-resolution structures of GPCR signaling complexes with various transducers, and especially the limited number and resolution of GPCR–arrestin structures, the intricate molecular mechanisms underlying the activation of G proteins and arrestins by GPCRs have remained largely elusive. Our high-resolution µOR–G_z_ and µOR–βarr1 structures enabled us to observe subtle conformational changes in key motifs of µOR compared with the µOR–G_i_ complex. In these three complexes, DAMGO and endomorphin-1 assume a generally comparable orientation within the μOR binding pocket; however, they differ in the details, particularly the conformation of Tyr1 (Supplementary information, Fig. S6a, b). Different ligands lead to conformational differences in key residues in the μOR binding pocket (Fig. 5a, c; Supplementary information, Fig. S6c, d). Most residues in the binding pocket adopt nearly identical side-chain conformations when μOR is bound to the three different transducers, except for the triadic polar network Q^2.60^D^3.32^Y^7.43^ and the toggle switch W293^6.48^ (Fig. 5b, c; Supplementary information, Fig. S6c, d). The DAMGO-activated signaling complex consists of a monomeric μOR and its associated signaling proteins, whereas the endomorphin-1–μOR–G_i_ complex forms antiparallel dimers.^23,28^ Consequently, our analysis of conformational changes focused on the structural transitions in the signaling complexes upon activation by DAMGO.

D147^3.32^ forms an H-bond with a water molecule (water^1^) in the polar channel of βarr1-coupled μOR (Fig. 5b-I; Supplementary information, Fig. S2). Beneath water^1^, there is a weak but discernible density corresponding to water^2^, which forms an H-bond bridge together with water^1^, thereby connecting the extracellular ligand-binding domain (LBD) to the polar network in the connector region of µOR (Fig. 5b, c; Supplementary information, Fig. S2). In our DAMGO–µOR–G_z_ map and the previously resolved DAMGO–µOR–G_i_ map (EMD-28088),^23^ water^1^ is absent; water^2^ mediates the interaction between S154^3.39^ and S329^7.46^ and is present in both maps, though it was not modeled in the latter. Compared with the G_i_- and G_z_-coupled µOR complexes, S329^7.46^ turns toward TM1 and TM2 in the βarr1-coupled μOR, forming H-bonds with N86^1.50^ and T118^2.54^. This rearrangement establishes two triadic polar networks in the connector region of µOR, namely D114^2.50^S154^3.39^N332^7.49^ and N86^1.50^T118^2.54^S329^7.46^, which stabilize activated µOR in the βarr1-coupled state (Fig. 5b; Supplementary information, Figs. S2, S6c–e).

Compared to G_i_-bound µOR, W293^6.48^ tilts upward by 0.7 Å and 1.2 Å in DAMGO-activated G_z_-coupled µOR and βarr1-coupled µOR, respectively; this is accompanied by a conformational alteration in N328^7.45^ that preserves the H-bond with W293^6.48^ (Fig. 5b-I; Supplementary information, Fig. S6c). In the G_i_-bound µOR, N328^7.45^ forms a 2.8-Å H-bond with N332^7.49^, which constrains W293^6.48^ and the N^7.49^PxxY^7.53^ motif. Due to the movement of W293^6.48^, the H-bond between N328^7.45^ and N332^7.49^ loosens, releasing N332^7.49^ to form a strong H-bond with the conserved residue D114^2.50^ in the G_z_-bound µOR (3.5 Å) and the βarr1-bound µOR (2.6 Å) (Fig. 5b; Supplementary information, Figs. S2, S6c, d).

Conformational transitions in W^6.48^ and the triadic polar network Q^2.60^D^3.32^Y^7.43^ display cascading effects that reshape the interaction network between TMs 1, 2, and 7, ultimately transmitting signals from the extracellular pocket to the N^7.49^PxxY^7.53^ motif (Fig. 5b, c). Within this motif, P333^7.50^ and Y336^7.53^ undergo coordinated rearrangements, resulting in conformational changes in the intracellular coupling domain (ICD), thereby enabling the accommodation of G_i_, G_z_, or βarr1 (Fig. 5b; Supplementary information, Fig. S6c, d). The α5 helices of G_i_ and G_z_, and the finger loop of βarr1, insert into the receptor intracellular pocket, driving TM7 and helix8 into state-specific conformations. This reconfigures the interaction between the intracellular end of TM1 and the TM2–TM7–helix8 interface, and the rearrangement propagates back to influence the extracellular LBD. In this cascade, TM1 acts as a key conformational regulator that links the extracellular and intracellular domains of µOR across different signaling complexes (Fig. 5c; Supplementary information, Fig. S6d). In summary, the dynamic plasticity of TM1 selectively stabilizes receptor conformations, thereby enabling preferential engagement with different signaling proteins.

### Activating μOR with DAMGO and endomorphin-1

**Fig. 6:**
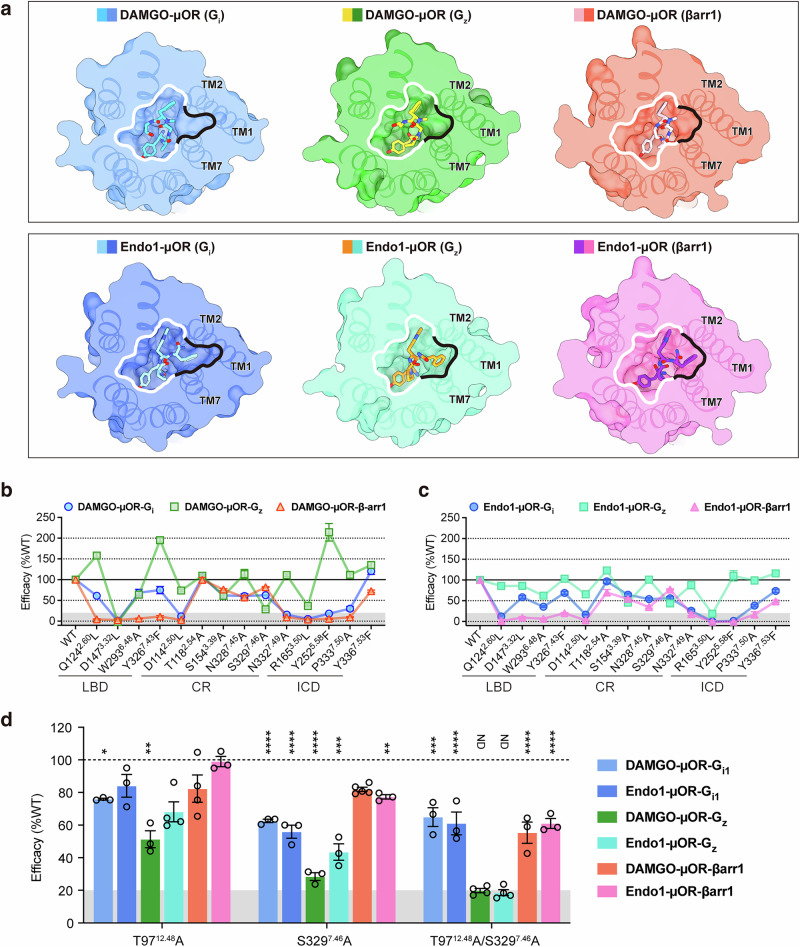
Comparisons of DAMGO and endomorphin-1 in activating μOR. **a** Comparison of the DAMGO- and endomorphin-1–binding pockets in μOR coupled to G_i_, G_z_, and βarr1. Structures are shown as surface slice representations to illustrate the formation of the ligand-binding pocket. **b**, **c** Mutagenesis analysis of the key residues in βarr1-, G_z_-, and G_i_-bound μOR activated by DAMGO (**b**) and endomorphin-1 (Endo1) (**c**), measured using a BRET assay. Data represent mean ± SEM of n ≥ 3 biological replicates. **d** Mutagenesis analysis of the key residues for G_z_ signaling in βarr1-, G_z_-, and G_i_-bound μOR activated by DAMGO and endomorphin-1 (Endo1), measured using a BRET assay. Data represent mean ± SEM of n ≥ 3 biological replicates.

The DAMGO- and endomorphin-1-activated μOR signaling complexes display similar conformations, including the ligand-binding pocket. In the µOR–βarr1 complex, the TM1-fusion pocket is shallower than in the µOR–G_i_ complex (Fig. 6a). Differential engagement of endomorphin-1, especially the insertion of Phe4 of endomorphin-1 into the TM1-fusion pocket, induces ligand-dependent conformational changes within µOR, resulting in divergent functional profiles driven by critical rearrangements of residues along the µOR activation pathway (Fig. 6a–c).

In the µOR–βarr1 complex, D147^3.32^ reorients in response to the positioning of DAMGO or endomorphin-1 to maintain the H-bond with Tyr1 of each ligand, consequently weakening its interaction with Y326^7.43^ (2.4 Å, 2.3 Å, and 3.7 Å in DAMGO–µOR–G_i_, –G_z_, and –βarr1, and 2.6 Å, 2.7 Å, and 3.4 Å in endomorphin-1–µOR–G_i_, –G_z_, and –βarr1, respectively) (Fig. 5b-I, c-I; Supplementary information, Fig. S6c-I, d-I). D147^3.32^ clearly plays a significant role in both the ligand binding and stabilization of µOR complexes (Fig. 6b, c; Supplementary information, Figs. S6c, d, S7a, b). Accordingly, the D147^3.32^L mutation completely abolished DAMGO-activated signaling of G_i_, G_z_, and βarr1 and significantly decreased endomorphin-1-activated signaling (Fig. 6b, c; Supplementary information, Fig. S7a, b and Tables S2–S4). The interaction between endomorphin-1 and the TM1-fusion pocket results in reduced dependence on D147^3.32^. Y326^7.43^ forms a stronger interaction with Q124^2.60^ in the DAMGO–µOR–βarr1 complex than in the other two complexes (3.2 Å, 2.9 Å, and 2.6 Å in G_i_-, G_z_-, and βarr1-bound µOR, respectively) (Fig. 5c–I). In contrast, this H-bond is weaker in the endomorphin-1–µOR–βarr1 complex (2.8 Å, 3.2 Å, and 3.5 Å in G_i_-, G_z_-, and βarr1-bound µOR, respectively) (Supplementary information, Fig. S6d-I). Nevertheless, the hydrogen-bonding interactions between Y326^7.43^ and Q124^2.60^ in the Q^2.60^D^3.32^Y^7.43^ triad are particularly significant in the µOR–βarr1 complex. Accordingly, the Q124^2.60^L mutation completely abolished βarr1 recruitment and maintained about 60% efficacy of G_i_ dissociation and 160% efficacy of G_z_ dissociation in the presence of DAMGO. In contrast, Q124^2.60^L nearly abolished βarr1 and G_i_ recruitment and maintained 85% efficacy of G_z_ activation in the presence of endomorphin-1 (Fig. 6b, c; Supplementary information, Fig. S7a, b and Tables S2–S4). The close interaction between endomorphin-1 and Q124^2.60^ has a greater effect on downstream µOR signaling than the corresponding interaction observed with DAMGO. The Y326^7.43^F mutation significantly reduced βarr1 recruitment efficacy by over 75% and moderately decreased G_i_ dissociation efficacy by 30%, yet it did not impair G_z_ dissociation efficacy. Surprisingly, the efficacy of DAMGO-induced G_z_ dissociation was twice that of the wild type, thereby uncovering a role for Y326^7.43^ in biased signaling regulation (Fig. 6b, c; Supplementary information, Fig. S7a, b and Tables S2, S3).

In GPCRs, position 6.48 is acknowledged as a critical switch during the activation process.^6,44^ Remarkably, the µOR toggle switch mutation W293^6.48^A eliminated βarr1 recruitment while retaining 35%–60% G_i_ activation efficacy and 60%–70% G_z_ activation efficacy (Fig. 6b, c; Supplementary information, Fig. S7a, b and Tables S2–S4), indicating that W293^6.48^ is crucial for triggering βarr1 signaling. In the DAMGO–µOR–βarr1 complex, DAMGO adopts a distinct conformation compared to that in the µOR–G_i_ complex: the side chain of Tyr1 reorients, allowing the oxygen atom to form a hydrogen-bonding network with H297^6.52^ through a water molecule (water^4^ absent in the µOR–G protein complexes and the endomorphin-1–µOR–βarr1 complex) (Supplementary information, Figs. S2, S7c). H297^6.52^ then transmits the signal to W293^6.48^, inducing a distinct conformational change at this site. Simultaneously, the upward tilt of W293^6.48^ toward DAMGO, compared to the G_i_-bound µOR, establishes a hydrophobic interaction with DAMGO (Supplementary information, Fig. S7c). This explains why W293^6.48^ has a more important role in βarr1 signaling than in G protein signaling. In the endomorphin-1–µOR–βarr1 complex, endomorphin-1 displays a markedly different conformation than that in the µOR–G_i_ complex: the side chain of Tyr1 turns toward TM3, allowing the oxygen atom to form a hydrogen-bonding network with Y148^3.33^ through a water molecule (water^1^, which is not observed in the µOR–G_i/z_ complex or the DAMGO–µOR–βarr1 complex) (Supplementary information, Figs. S2, S6c–I, S7c). The different conformation of endomorphin-1 also leads to an upward shift of W293^6.48^ in µOR compared to the endomorphin-1–µOR–G_i_ complex (Supplementary information, Fig. S6c-I). Although the reasons for the ligand-induced changes at position 6.48 differ, the ultimate effect in both cases is an upward shift of position 6.48, mediating signal transduction from the LBD to the ICD. Indeed, H297^6.52^ and Y148^3.33^ have different effects on the ability of the two ligands to activate µOR. Specifically, H297^6.52^ has a greater effect on DAMGO-induced µOR activation, whereas Y148^3.33^ has a greater effect on endomorphin-1-induced µOR activation (Supplementary information, Fig. S7d and Tables S2–S4). Consequently, the conformational changes of W293^6.48^ and the triadic polar network Q^2.60^D^3.32^Y^7.43^ transmit signals from the ligand-binding pocket to N332^7.49^ in the N^7.49^PxxY^7.53^ motif, which is adjacent to the intracellular pocket; this process is accompanied by a series of polar network rearrangements in µOR (Fig. 5; Supplementary information, Fig. S6c, d).

Among residues in the connector region, D114^2.50^ and N332^7.49^ had the most significant effects on receptor activation; in particular, they nearly abolished the G_i_ and βarr1 signaling pathways. Mutations of other residues in the polar network had little effect on the potency of receptor activation but resulted in reduced signaling efficacy; G_z_ signaling was typically the least affected. Notably, S329^7.46^A decreased βarr1 recruitment efficacy by ~20% but reduced G_i_ and G_z_ dissociation efficacy by 40% and 80%, respectively (Fig. 6b, c; Supplementary information, Fig. S7a, b and Tables S2–S4). The relatively minor effect of the S329^7.46^ mutation on βarr1 recruitment may stem from the inward shift of TM1 in βarr1-bound μOR, which leads to a more compact and stable interaction among TMs 1, 2, and 7. The mutation at position 7.46 notably diminished the efficacy of G protein signaling, which may be attributed to the critical role of this residue in the internal polar network of the connector region during G protein signaling. Specifically, S329^7.46^ is pivotal in stabilizing the triadic polar network D^2.50^S^3.39^N^7.49^, which is required for the proper transmission of G_z_ signaling (Fig. 5b, c). In the ICD, Y252^5.58^ and R165^3.50^ form a hydrogen-bonding network that stabilizes the intracellular pocket in the activated state (Fig. 5b-IV; Supplementary information, Fig. S6a-IV). Mutations in these residues had a significant effect on μOR signaling (Fig. 6b, c; Supplementary information, Fig. S7a, b and Tables S2–S4). In the βarr1–μOR complex, an H-bond forms between Y252^5.58^ and Y336^7.53^ (3.6 Å, 3.8 Å, and 4.0 Å in βarr1–, G_z_–, and G_i_–μOR, respectively). Accordingly, mutation of Y326^7.43^ resulted in the greatest decrease in βarr1 signaling efficacy (up to 30%–50%) but minimally affected G_i/z_ signaling. P333^7.50^A had the greatest effect on βarr1 and a lesser effect on G_i/z_ signaling (Fig. 6b, c; Supplementary information, Fig. S7a, b and Tables S2–S4), suggesting that βarr1 signaling is more dependent on the stability of the μOR intracellular pocket.

In the wild-type and most of the mutant constructs, G_z_ exhibited strong signaling. However, in our mutagenesis study, T97^12.48^A and S329^7.46^A showed a considerable reduction in G_z_ signaling. Furthermore, introduction of the double mutation T97^12.48^A/S329^7.46^A nearly abrogated G_z_ signaling (Fig. 6d; Supplementary information, Tables S2–S4). This double mutant, with its reduced G_z_ signaling, may facilitate G_z_-dependent pathophysiological studies and provide a clearer direction for µOR-targeted drug development.

Overall, the LBD exhibits varying effects upon µOR activation by different agonists. Nevertheless, the critical residues in the connector region and ICD that are implicated in the activation cascade assume similar conformations and functional attributes. This suggests a conserved signal transduction mechanism across diverse μOR agonists.

### The mechanism of biased signal transduction in μOR

**Fig. 7:**
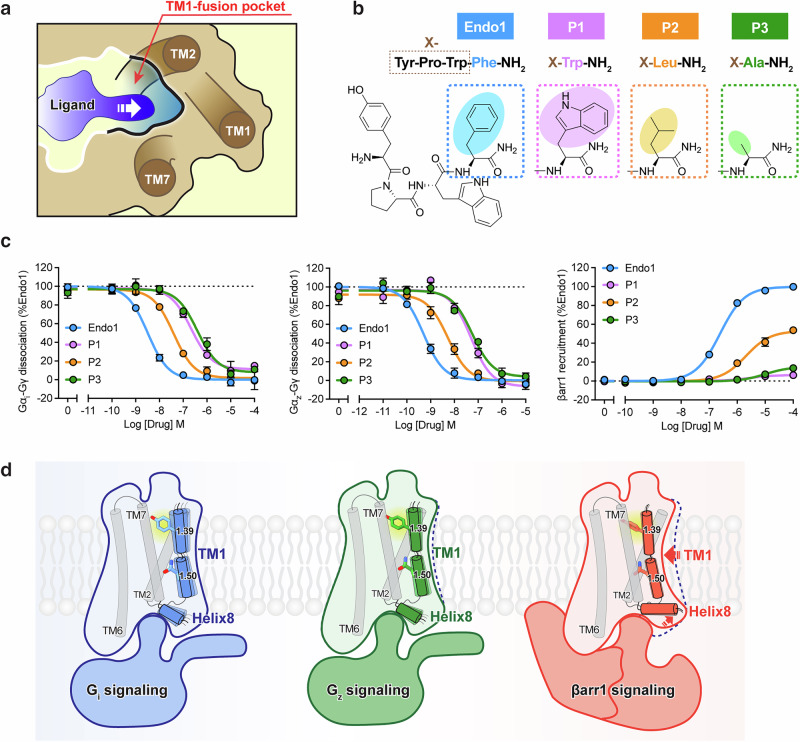
Molecular mechanism of μOR-mediated signal transduction. **a** Schematic illustration of the extracellular TM1-fusion pocket of μOR, formed by TMs 1, 2, and 7. **b** Sequence of endomorphin-1 (Endo1) and modified peptide ligands. P1–P3 correspond to mutations of phenylalanine 4 (Phe4) to tryptophan (Trp), leucine (Leu), and alanine (Ala), respectively. **c** Functional assays showing peptide-induced activation of G_i_-, G_z_-, and βarr1-mediated μOR signaling, measured using a BRET assay. Data represent mean ± SEM of n ≥ 3 biological replicates. **d** Model of DAMGO-bound μOR in the G_i_-coupled (cornflower blue), G_z_-coupled (forest green), and βarr1-coupled (tomato) states. Essential residues and crucial regions are marked with different colors and patterns. Red arrows indicate the movements of μOR helices in the βarr1-coupled state relative to the G_i_-coupled state.

In the μOR binding pocket, endomorphin-1 occupies an additional site compared to DAMGO — the TM1-fusion pocket, formed by TMs 1, 2, and 7. The Phe4 of endomorphin-1 fits into this pocket and participates in the dynamic regulation of downstream signaling by TM1 (Fig. 7a). We hypothesized that replacing Phe4 with a residue possessing a larger side chain might restrict the internal movement of TM1, thereby affecting βarr1 signaling. Therefore, we designed variant peptides (P1–P3) and evaluated their effect on downstream signal transduction (Fig. 7b). Substitution of Phe4 with tryptophan (P1) abolished βarr1 signaling but retained partial G_i/z_ signaling, substitution with leucine (P2) had minimal effects on downstream signaling, and substitution with alanine (P3) slightly decreased G_i/z_ signaling but abolished βarr1 signaling (Fig. 7c; Supplementary information, Table S5). Reduction in the size of the side chain at this position of endomorphin-1 may affect the stability of the TM1-fusion pocket. Although DAMGO lacks a side chain that inserts into the TM1-fusion pocket, the two residues between Tyr1 and Phe4 are close to TMs 2 and 7, which may induce DAMGO’s central region to bend toward the TM2–TM7 interface (Supplementary information, Fig. S7c). This conformational change may compensate for the absence of side chain insertion into the TM1-fusion pocket. Through characterization of the TM1-fusion pocket, we identified a new strategy for ligand modification that may facilitate drug development targeting enhanced selectivity for downstream signaling pathways.

The structures of antagonist-bound and agonist-bound μOR in the G_i_-, G_z_-, and βarr1-coupled states have provided important insights into the molecular mechanism by which μOR activates different transducers. Upon agonist binding, μOR’s extracellular TM bundles are drawn together, resulting in repositioning of the triadic polar network Q^2.60^D^3.32^Y^7.43^ and the toggle switch. In the G protein- or βarr1-coupled state, the side chain of W293^6.48^ rotates downward, accompanied by outward swinging of TM6 and the subsequent opening of the intracellular pocket (Supplementary information, Fig. S3b). Upon µOR activation, in addition to the opening of TM6, engagement with downstream signaling proteins is further regulated by TM1 and helix8. The dynamic movements of TM1 (inward/outward) and helix8 (upward/downward) alter the interactions at the TM2–TM7 interface, including those of Y75^1.39^ with the triadic polar network Q^2.60^D^3.32^Y^7.43^, those of N^1.50^ with D^2.50^ and S^7.46^, and those of the intracellular end of TM1 with helix8 and N^7.49^PxxY^7.53^, thereby fine-tuning µOR’s selectivity for downstream signaling proteins (Fig. 7d). The structural elucidation and in-depth analysis of μOR activation mechanisms enhance the understanding of agonist structure–activity relationships across diverse transducers and provide insights for the structure-based discovery of biased agonists targeting μOR.

## Discussion

We resolved high-resolution structures of μOR in complex with its two major effector proteins, G_z_ and βarr1. Together with the μOR–G_i_ complex, these structures establish a complete structural framework for understanding the biased activation of downstream signaling pathways by μOR. Structural insights, combined with MD simulations and comprehensive mutagenesis studies, reveal the conformational selectivity of μOR in its interactions with downstream transducers. Critically, we demonstrated the pivotal role of TM1 dynamics in modulating G_i_-, G_z_-, and βarr1-mediated μOR signaling. Peptides based on endomorphin-1, combined with signal transduction experiments, further elucidated the involvement of the TM1-fusion pocket in regulating μOR coupling to G_i_, G_z_, and βarr1. Collectively, these findings provide compelling evidence of the interplay between TM1 conformational dynamics and downstream signal transduction pathways and carry implications for the regulatory mechanisms of GPCRs.

TM1 is positioned at the outer edge of the GPCR transmembrane bundle, away from both the extracellular ligand-binding pocket and the intracellular signaling protein-binding pocket. Therefore, its regulatory roles in GPCR ligand binding and effector protein coupling are often overlooked. Extending from the extracellular to the intracellular regions, TM1 may broadly and dynamically regulate the ligand binding and signal transduction of GPCRs. Our investigation of TM1’s dynamic regulation of µOR signaling advances the understanding of the intrinsic conformational dynamics and functional modulation of GPCRs and may also be applicable to other receptors within the superfamily.

GPCRs exhibit diverse oligomeric configurations, including homomeric and heteromeric assemblies, and can also associate with non-GPCR transmembrane proteins to form multiprotein complexes.^45,46^ Interactions between the TMDs of these proteins induce conformational alterations in the transmembrane regions of GPCRs, thereby modulating receptor activation and downstream signal transduction. It is well established that class C GPCRs are obligate dimers, composed of two identical or homologous subunits.^47^ Different GPCRs exhibit distinct dimerization interfaces, occasionally with TM1 participating in interface formation. The interface of the fungal class D1 GPCR Ste2 homodimer is primarily composed of the N-terminus, ECL1, TM1, and TM7.^48^ In addition, TM1 has been implicated in the formation of dimeric interfaces in several class A GPCRs. The role of TM1 in dimer interface assembly has been visually demonstrated in the X-ray crystal structures of β_1_AR, µOR, and κOR.^26,49,50^ Cysteine cross-linking techniques have further clarified TM1’s role in establishing the dimer interface of the platelet-activating factor receptor,^51^ and MD simulations have suggested that the TM1-mediated dimer interface in the CCR5 homodimer is stable.^52^ By combining double electron–electron resonance (DEER), single-molecule fluorescence resonance energy transfer (smFRET), and MD simulations, researchers have proposed models for dimer interfaces involving TM1 that may facilitate G protein activation in NTSR1.^53^ The involvement of TM1 in the dimerization process of certain GPCRs suggests that TM1 may play a regulatory role in the downstream signaling of these receptors.

Several non-GPCR transmembrane proteins also function as interaction partners that bind to the transmembrane domains of GPCRs to modulate their activity. Notable examples include receptor activity-modifying proteins (RAMPs), which interact with at least 11 different GPCRs.^54^ For instance, co-expression of RAMPs with the calcitonin receptor (CTR) alters the ligand specificity of CTR.^55^ Similarly, the transmembrane helix of the CD69 homodimer interacts with TM4 of the sphingosine 1-phosphate receptor 1 (S1PR1), inducing an allosteric shift in S1PR1 TMs 5–6 that directly activates the receptor and enables engagement with heterotrimeric G_i_.^56^ Furthermore, the melanocortin 2 receptor, facilitated by association with the transmembrane protein melanocortin receptor accessory protein 1 (MRAP1) adjacent to TMs 4–5, responds to adrenocorticotropic hormone to promote glucocorticoid biosynthesis and cortisol secretion.^57^ Therefore, proteins binding to the TM bundle of GPCRs can influence receptor activity. In general, the TM helices of GPCRs participate in the dynamic conformational changes and signaling processes of GPCRs. TMs 2–3 and 5–7, typically located in the receptor core region where ligands and effectors bind, have received considerable attention for their dynamic variations and regulatory roles in signal transduction. In contrast, TM1 and TM4 typically do not directly participate in ligand or effector protein binding. Consequently, their roles in the signaling process remain poorly characterized. Our findings, in addition to the above examples, suggest that peripheral transmembrane helices also contribute to the regulation of ligand binding and signal transduction in GPCRs. RAMPs that engage TM1 may be identified through further research supported by artificial intelligence.

## Materials and methods

### Constructs

Wild-type mouse *OPRM1*, fused to an N-terminal hemagglutinin (HA) signal sequence and Flag tag and to a C-terminal 3C protease cleavage site, 2× MBP tag, and 10× His tag, was cloned into a modified pFastBac baculovirus expression vector (Invitrogen). The LgBiT sequence was fused to the C-terminus of μOR to enhance the stability of the μOR–G_z_ complex. The C-terminal tail of μOR (residues 353–398) was replaced with the C-terminus of the vasopressin 2 receptor to enhance the stability of the μOR–βarr1 complex. The human dominant-negative DNGα_z_-coding sequence was cloned into the pFastBac vector and modified by site-directed mutagenesis, as previously described, to decrease nucleotide-binding affinity and increase G protein stability.^29^ Specifically, the N-terminal sequence of Gα_z_ was replaced with the G_i1_ sequence (residues 1–29) to allow for better interaction with scFv16; other mutations included S47N, G204A, E246A, R249K, N258K, N262D, and A327S. Human Gβ1- and Gγ2-coding sequences were cloned into the pFastBac-Dual vector, with Gβ1 fused to a C-terminal SmBiT (peptide 86, Promega). The *Bos taurus* βarr1-coding sequence was cloned into the pFastBac vector with a truncation at residue 394, an R169E mutation, and the 3 A mutations (I386A, V387A, and F388A); these modifications remove autoinhibition, resulting in a constitutively active state and enhanced complex stability.

For the βarr1 recruitment assay, full-length mouse *OPRM1* with an N-terminal Flag tag and a C-terminal RLuc8 tag (wild-type or mutant μOR-RLuc8) and *ARRB1* with an N-terminal Venus tag (Venus-βarr1) were each cloned into the pcDNA3.1 plasmid. For the G_i_/G_z_ dissociation assay, *OPRM1* with an N-terminal Flag tag (wild-type or mutant μOR) was cloned into pcDNA3.1. The N-terminal Flag tag was used for subsequent enzyme-linked immunosorbent assay (ELISA) assays of μOR expression levels. Gα_i_ and Gα_z_ were fused with the RLuc8 fragment, which was inserted after Leu91 in Gα_i1_ and after Pro114 in Gα_z_ (Gα_i1_-RLuc8 and Gα_z_-RLuc8).^18^ The Venus fragment was N-terminally fused to a Gγ2(C68S) subunit using a flexible 15-amino-acid linker (Venus-Gγ2). For the G protein dissociation assay, the coding sequences of Gα_i1_-RLuc8, Gα_z_-RLuc8, Venus-Gγ2, and Gβ1 were cloned into pcDNA3.1. All constructs were confirmed by sequencing.

### Expression and purification of scFv16

The scFv16 protein was expressed and purified using the Bac-to-Bac system as previously described.^25^ In brief, the scFv16-coding sequence with an N-terminal GP67 signal peptide and a C-terminal 8× His tag was subcloned into the pFastBac vector. The protein was expressed and secreted into the culture medium by *Trichoplusia ni* Hi5 insect cells for 48 h. The cell culture supernatant was collected and scFv16 was purified using Ni-NTA resin via affinity chromatography. The eluted protein was concentrated and loaded onto a HiPrep 26/10 desalting column (GE Healthcare) with a running buffer containing 20 mM HEPES (pH 7.5) and 100 mM NaCl. The monomeric fractions were pooled, then the purified scFv16 was concentrated, flash-frozen in liquid nitrogen, and stored at −80 °C until use.

### Expression and purification of Fab30

Fab30 with a C-terminal 9× His tag was expressed in the periplasm of *Escherichia coli* BL21(DE3) cells. Cells containing the recombinant plasmid were cultured in 2× YT medium at 37 °C until OD_600_ reached 1.0. Expression was induced with 1 mM IPTG, and cultures were grown at 37 °C for 5 h. Cells were harvested by centrifugation at 3000× *g* for 15 min and subsequently lysed in lysis buffer (500 mM Sucrose, 200 mM Tris-HCl (pH 8.0), 0.5 mM EDTA, 1 mM PMSF, 0.5 mg/mL lysozyme, 0.01 mg/mL DNase I) with stirring at room temperature (RT) for 30 min. Proteins were purified using Ni-NTA resin.^33^ The eluted protein was concentrated and loaded onto a HiPrep 26/10 desalting column (GE Healthcare) with a running buffer containing 20 mM HEPES (pH 7.5), 100 mM NaCl, and 10% (w/v) Glycerol. The monomeric fractions were pooled, then the purified Fab30 was concentrated, flash-frozen in liquid nitrogen, and stored at −80 °C until use.

### Complex formation and purification

*Spodoptera frugiperda* (Sf9; Expression Systems) cells cultured in ESF 921 medium (Expression Systems) were used for protein expression. After growth to a density of 2.2 × 10^6^ cells/mL, Sf9 cells were infected with baculoviruses carrying μOR, Gα_z_, and Gβ1/γ2 at a 1:4:1 ratio for expression of the μOR–G_z_ signaling complex, or with baculoviruses carrying μOR, βarr1, and GRK2/5 at a 1:1:1 ratio for expression of the μOR–βarr1 signaling complex. The infected cells were cultured at 27 °C for 48 h and collected by centrifugation, then the cell precipitates were flash-frozen in liquid nitrogen and stored at −80 °C until further use.

For purification of the μOR–G_z_ and μOR–βarr1 complexes, the cell pellets were thawed on ice, suspended, and lysed by Dounce homogenization in a buffer containing 20 mM HEPES (pH 7.5), 150 mM NaCl, and 2 mM MgCl_2_ supplemented with EDTA-free protease inhibitor cocktail (Bimake). μOR–G_z_ complex formation was initiated using 2.5 mg scFv16 antibody, 12 mU/mL apyrase (Sigma), and the agonist (10 μM DAMGO or 100 μM endomorphin-1). μOR–βarr1 complex formation was initiated using 20 μg/mL Fab30 and the agonist (10 μM DAMGO or 100 μM endomorphin-1). The mixture was incubated for 1 h at RT and then solubilized with 0.5% (w/v) lauryl maltose neopentyl glycol (LMNG, Anatrace) and 0.1% (w/v) cholesterol hemisuccinate (CHS, Anatrace) for 2 h at 4 °C. After centrifugation at 30,000× *g* for 30 min at 4 °C, the supernatant was isolated and incubated with amylose resin (NEB) for 1 h at 4 °C. The protein was eluted with 10 mM maltose, treated with 3C protease for 1 h at RT to remove the 2× MBP tag, concentrated using a 100-kDa cutoff concentrator (Millipore), and loaded onto a Superose 6 Increase 10/300 GL column (GE Healthcare) with a running buffer containing 20 mM HEPES (pH 7.5), 150 mM NaCl, 2 mM MgCl_2_, 0.00075% (w/v) LMNG, 0.0002% (w/v) CHS, 0.00025% (w/v) GDN (Anatrace), and the agonist (1 μM DAMGO or 1 μM endomorphin-1). Fractions corresponding to the monomeric complex were collected and concentrated for electron microscopy experiments.

### Cryo-EM grid preparation and data collection

First, the DAMGO–μOR–G_z_ complex, DAMGO–μOR–βarr1 complex, endomorphin-1–μOR–G_z_ complex, and endomorphin-1–μOR–βarr1 complex were concentrated to about 6 mg/mL, 15 mg/mL, 12 mg/mL, and 15 mg/mL, respectively. For cryo-EM grid preparation, about 3 μL of each purified complex was individually applied to glow-discharged holey carbon grids (Quantifoil, R1.2/1.3, 300 mesh). The grids were blotted for 3.5 s with a blot force of 10 at 4 °C and 100% humidity, then plunge-frozen in liquid ethane using a Vitrobot Mark IV (Thermo Fisher Scientific). Cryo-EM data collection was performed on a Titan Krios microscope (FEI) operated at 300 kV accelerating voltage at the Center of Cryo-Electron Microscopy (Liangzhu Laboratory). Micrographs were acquired using a CFEG, a Selectris energy filter, and a Falcon 4 direct electron detector. Automated data collection was carried out using EPU software according to standard procedures. Imaging was performed at a magnification of 130,000×, yielding a pixel size of 0.93 Å. The defocus range was set from −0.6 to −2.0 μm. Each micrograph was dose-fractionated into 40 frames at a dose rate of 7.49 e^−^ per pixel per second, with a total exposure time of 6 s, resulting in a total dose of about 52 e^−^ Å^−2^. A total of 7680 movies were collected for the DAMGO–μOR–G_z_ complex, 48,893 for the DAMGO–μOR–βarr1 complex, 6518 for the endomorphin-1–μOR–G_z_ complex, and 14,940 for the endomorphin-1–μOR–βarr1 complex.

### Cryo-EM data processing

Movies were aligned using Relion’s implementation of the MotionCor2 algorithm. Contrast transfer function (CTF) parameters were estimated using Gctf v1.18. Cryo-EM data processing was performed with Relion 4.0 and CryoSPARC v4.0.3.

For the DAMGO–μOR–G_z_–scFv16 complex, template-based particle selection yielded 5,149,386 particle projections in Relion. The projections were imported into CryoSPARC for several rounds of 2D classification. The selected particle images were subjected to ab initio reconstruction to generate initial reference maps, followed by several rounds of heterogeneous refinement in CryoSPARC. The resulting subset of 577,846 particle projections was re-extracted and subjected to two rounds of 3D classification in Relion to further remove particles in poorly defined classes. The final subset of 136,606 particle projections was subjected to 3D refinement, CTF refinement, and Bayesian polishing in Relion, generating a map with a global resolution of 2.8 Å.

For the DAMGO–μOR–βarr1–Fab30 complex, template-based particle selection yielded 38,555,092 particle projections in Relion. The projections were imported into CryoSPARC for several rounds of 2D classification. The selected particle images were subjected to ab initio reconstruction to generate initial reference maps, followed by several rounds of heterogeneous refinement in CryoSPARC. The resulting subset of 1,131,027 particle projections was re-extracted and subjected to two rounds of 3D classification in Relion to further remove particles in poorly defined classes. The final subset of 233,269 particle projections was subjected to 3D refinement, CTF refinement, and Bayesian polishing in Relion, generating a map with a global resolution of 2.8 Å.

For the endomorphin-1–μOR–G_z_–scFv16 complex, template-based particle selection yielded 2,791,125 particle projections in Relion. The projections were imported into CryoSPARC for several rounds of 2D classification. The selected particle images were subjected to ab initio reconstruction to generate initial reference maps, followed by several rounds of heterogeneous refinement in CryoSPARC. The resulting subset of 648,889 particle projections was re-extracted and subjected to two rounds of 3D classification in Relion to further remove particles in poorly defined classes. The final subset of 149,926 particle projections was subjected to 3D refinement, CTF refinement, and Bayesian polishing in Relion, generating a map with a global resolution of 2.8 Å.

For the endomorphin-1–μOR–βarr1–Fab30 complex, template-based particle selection yielded 11,628,463 particle projections in Relion. The projections were imported into CryoSPARC for several rounds of 2D classification. The selected particle images were subjected to ab initio reconstruction to generate initial reference maps, followed by several rounds of heterogeneous refinement in CryoSPARC. The resulting subset of 879,582 particle projections was re-extracted and subjected to two rounds of 3D classification in Relion to further remove particles in poorly defined classes. The final subset of 206,690 particle projections was subjected to 3D refinement, CTF refinement, and Bayesian polishing in Relion, generating a map with a global resolution of 2.8 Å.

### Model building and refinement

The structures of DAMGO-bound μOR (PDB: 6dde) and M2R–βarr1–Fab30 (PDB: 6u1n) were used to generate the initial template. Models were manually docked into the density map with UCSF Chimera 1.16 (https://www.cgl.ucsf.edu/chimera/). The docked models were then subjected to flexible fitting in Rosetta, followed by iterative rounds of manual building in Coot and real-space refinement in Phenix 1.19.2 (https://phenix-online.org/). The final refinement statistics were validated using the “comprehensive validation (cryo-EM)” module in Phenix. Refinement statistics are provided in Supplementary information, Table S1. Structural figures were created using the UCSF ChimeraX 1.17 (https://www.cgl.ucsf.edu/chimerax/).

### Bioluminescence resonance energy transfer assays (BRET)

HEK293T cells were seeded in a six-well plate and grown to 80% confluence before transfection. For the μOR-mediated G_i_/G_z_ dissociation assay, HEK293T cells were co-transfected with mouse μOR, Gα_i_-RLuc8 or Gα_z_-RLuc8, Venus-Gγ2, and Gβ1 in a 3:1:2:2 ratio using Hieff Trans liposomal transfection reagent (Yeasen). For the μOR-mediated βarr1 recruitment assay, HEK293T cells were co-transfected with μOR-RLuc8 and Venus-βarr1 in a 1:6 ratio. After 6 h of transfection, cells were plated onto a 96-well plate treated with a cell adherent reagent (Applygen). The next day, cells were washed with D-Hank’s balanced salt solution (HBSS, Solarbio) to remove the complete medium, then incubated with 45 μL assay buffer (1× HBSS, 20 mM HEPES (pH 7.5), 0.1% (w/v) BSA) per well for 30 min at 37 °C. Next, the assay buffer was removed, and 45 μL of 5 μM coelenterazine-h (Maokangbio) in assay buffer was added to each well, followed by incubation for 5 min at RT to allow for substrate diffusion. Luminescence was measured at 460–485 nm and fluorescent eYFP emission at 520–560 nm using a Spark Multimode Microplate Reader (Tecan). The eYFP/RLuc ratio was calculated as a baseline for each well. Ligands (5 μL of DAMGO, endomorphin-1, or modified peptides) were added at different concentrations and a second measurement was taken. The second eYFP/RLuc ratio was normalized to the baseline and analyzed in GraphPad Prism 9 (GraphPad Software Inc., San Diego, CA). Mutagenesis data were normalized to wild-type responses, and modified peptide stimulation data were normalized to the responses elicited by endomorphin-1. These data were then reanalyzed using nonlinear regression (“log(agonist) vs response”) in GraphPad Prism 9. The span and pEC_50_ values are provided in Supplementary information, Tables S3–S5.

### Cell-surface ELISA

Transfected cells were washed with 1× PBS and fixed with 4% paraformaldehyde for 10 min. Following fixation, cells were washed three times with 1× PBS and blocked with blocking buffer (1% (w/v) BSA/PBS) for 1 h at RT. Cells were then incubated with a 1:10,000 dilution of anti-FLAG M2 HRP-conjugated monoclonal antibody (Sigma-Aldrich, catalog number A8592; mouse IgG1) in blocking buffer for 0.5 h at RT. Wells were washed three times with blocking buffer and three times with 1× PBS. 80 μL working solution (mixed liquid of 7.5 μL luminol enhancer solution and 7.5 μL stable peroxide solution in 65 μL 1× PBS) containing SuperSignal ELISA Femto Maximum Sensitivity Substrate (Thermo Fisher Scientific) was added to each well. Luminescence was measured with a Spark Multimode Microplate Reader (Tecan) to assess receptor expression. Finally, data were normalized to the wild-type μOR signal (set as 100%) using GraphPad Prism 9. Expression levels of the mutant receptors are provided in Supplementary information, Table S2.

### B-factor analysis

For the atomic models of the DAMGO–μOR–G_z_, DAMGO–μOR–βarr1, endomorphin-1–μOR–G_z_, and endomorphin-1–μOR–βarr1 complexes, B-factors of individual residues were refined during the final real-space refinement in Phenix. In DAMGO-activated μOR–G_i_ (PDB: 8efq)/G_z_/βarr1 and endomorphin-1-activated μOR–G_z_/βarr1 structures, I155 in μOR is relatively stable and exhibits a low B-factor. Therefore, in each structure, its B-factor was set to 0 Å^2^ as a reference point, and the B-factors of all other residues were normalized by subtracting the refined B-factor of I155. Normalized B-factors of TM1 residues (T70–I93) were analyzed for the DAMGO–μOR–G_i_ (PDB: 8efq), DAMGO–μOR–G_z_, and DAMGO–μOR–βarr1 complexes. B-factors were also analyzed for monomeric μOR–G_i_ complexes activated by different agonists, including DAMGO (PDB: 6dde), lofentanil (LFT; PDB: 7t2h), C5 guanidinonaltrindole (PDB: 7u2l), C6 guanidinonaltrindole (PDB: 7u2k), PZM21 (PDB: 7sbf), FH210 (PDB: 7scg), TRV130 (PDB: 8efb), and mitragynine pseudoindoxyl (MP; PDB: 7t2g). B-factor coloring was generated using UCSF ChimeraX.

### RMSD measurement

RMSDs were calculated for pairwise comparisons among the DAMGO–μOR–G_i_/G_z_/βarr1 and endomorphin-1–μOR–G_i_/G_z_/βarr1 structures using the *rmsd sel* command in Chimera.

### Molecular dynamics simulations

The DAMGO–μOR–G_i_ (PDB: 8efq), DAMGO–μOR–G_z_, DAMGO–μOR–βarr1, endomorphin-1–μOR–G_z_, and endomorphin-1–μOR–βarr1 complexes were completed via homology modeling with the MODELLER program^58^ (Supplementary information, Fig. S4h-I). Protonation states were determined using H++^59^ (Supplementary information, Fig. S4h-I). All structures were embedded into an asymmetric lipid bilayer representing the plasma membrane, which was generated in CHARMM-GUI^60^ (Supplementary information, Fig. S4h-I). The outer leaflet of the lipid bilayer consisted of 33.3 mol% POPC, 33.3 mol% PSM, and 33.3 mol% cholesterol, whereas the inner leaflet consisted of 35 mol% POPC, 25 mol% POPE, 20 mol% POPS, and 20 mol% cholesterol. The total numbers of bilayer membrane lipids for the DAMGO–μOR–G_i_, DAMGO–μOR–G_z_, DAMGO–μOR–βarr1, endomorphin-1–μOR–G_z_, and endomorphin-1–μOR–βarr1 systems were 492, 492, 527, 539, and 489, respectively. The systems were then solvated in TIP3P water containing 0.15 M NaCl and neutralized with Cl^−^^61^ (Supplementary information, Fig. S4h-I). The dimensions of the simulation boxes were 11.8 × 11.8 × 17.3 nm^3^, 11.8 ×  11.8 × 16.8 nm^3^, 12.3 × 12.3 × 15.4 nm^3^, 12.8 × 12.8 × 16.9 nm^3^, and 12.3 × 12.3 × 15.7 nm^3^, respectively. The CHARMM36m force field was used to model the systems, whereas the CHARMM general force field (CGenFF) parameters were used to describe the ligands.^62^ MD simulations were conducted using GROMACS 2019.4.^63^ After 5000 steps of energy minimization with the steepest descent algorithm, a 250-ps NVT equilibration simulation and a cumulative 1.625-ns NPT equilibration to 1 atm were performed^64^ (Supplementary information, Fig. S4h-II). Long-range electrostatic interactions were treated using the particle-mesh Ewald method.^65^ Short-range electrostatic interactions and van der Waals interactions used a cutoff distance of 10 Å. All bonds were constrained using the LINCS algorithm.^66^ To avoid uncertainties in the sampling results, three 1000-ns production MD simulations with different initial velocities were performed for each system. System stability was evaluated using the RMSD for all of the heavy atoms in the ligand (DAMGO or endomorphin-1) and receptor; all simulation systems reached equilibrium within 50 ns (Supplementary information, Fig. S4h-III). The initial 50 ns were discarded, and only the final 950 ns of MD simulation data were used for subsequent analyses, including the RMSD calculation of TM1 after structural alignment based on the Cα atoms of TMs 2–7 (Fig. 2f; Supplementary information, Fig. S4i) and measurement of the distance between residues I278^6.33^ and E341^8.48^ (Fig. 3c). In GROMACS, the *rms* and *dist* functions were used for RMSD and distance calculations, respectively.

### Statistical analysis

Statistical analyses were performed on at least three different datasets using GraphPad Prism software. Data are presented as mean ± SEM from at least three independent experiments performed in triplicate (ns, not significant; ^ns^*P*  >  0.05; *P  <  0.05; **P  <  0.01; ***P  <  0.001; ****P  <  0.0001). One-way analysis of variance (ANOVA) was performed followed by Dunnett’s test, with comparisons made to the wild-type for mutagenesis data, to endomorphin-1 stimulation for modified peptides, and pairwise for B-factor data. For dose–response experiments, data were normalized and analyzed via nonlinear curve fitting with a three-parameter “log(agonist) vs response” model in GraphPad Prism 9.

## Supplementary information

Supplementary information, Figure S1

Supplementary information, Figure S2

Supplementary information, Figure S3

Supplementary information, Figure S4

Supplementary information, Figure S5

Supplementary information, Figure S6

Supplementary information, Figure S7

Supplementary information, Table S1

Supplementary information, Table S2

Supplementary information, Table S3

Supplementary information, Table S4

Supplementary information, Table S5

## Data Availability

The atomic coordinates and the electron microscopy maps of the DAMGO–μOR–βarr1, DAMGO–μOR–G_z_, endomorphin-1–μOR–βarr1 and endomorphin-1–μOR–G_z_ complexes have been deposited in the Protein Data Bank (PDB) and Electron Microscopy Data Bank (EMDB), respectively, under accession numbers: 9WSV/EMD-66207 (DAMGO–μOR–βarr1), 9WST/EMD-66205 (DAMGO–μOR–G_z_), 9WSX/EMD-66209 (endomorphin-1–μOR–βarr1), and 9WSW/EMD-66208 (endomorphin-1–μOR–G_z_). All relevant data are included in the manuscript or Supplementary Information.
