# Supplementary material for: The molecular basis of μ-opioid receptor signaling plasticity
Source: Cell Res. 2025 Nov 7;35(12):1021–36. doi: 10.1038/s41422-025-01191-8 (PMC12689640; doi:10.1038/s41422-025-01191-8)
Supplement: Supplementary file 1 — Supplementary information, Figure S1 [file 41422_2025_1191_MOESM1_ESM.pdf]

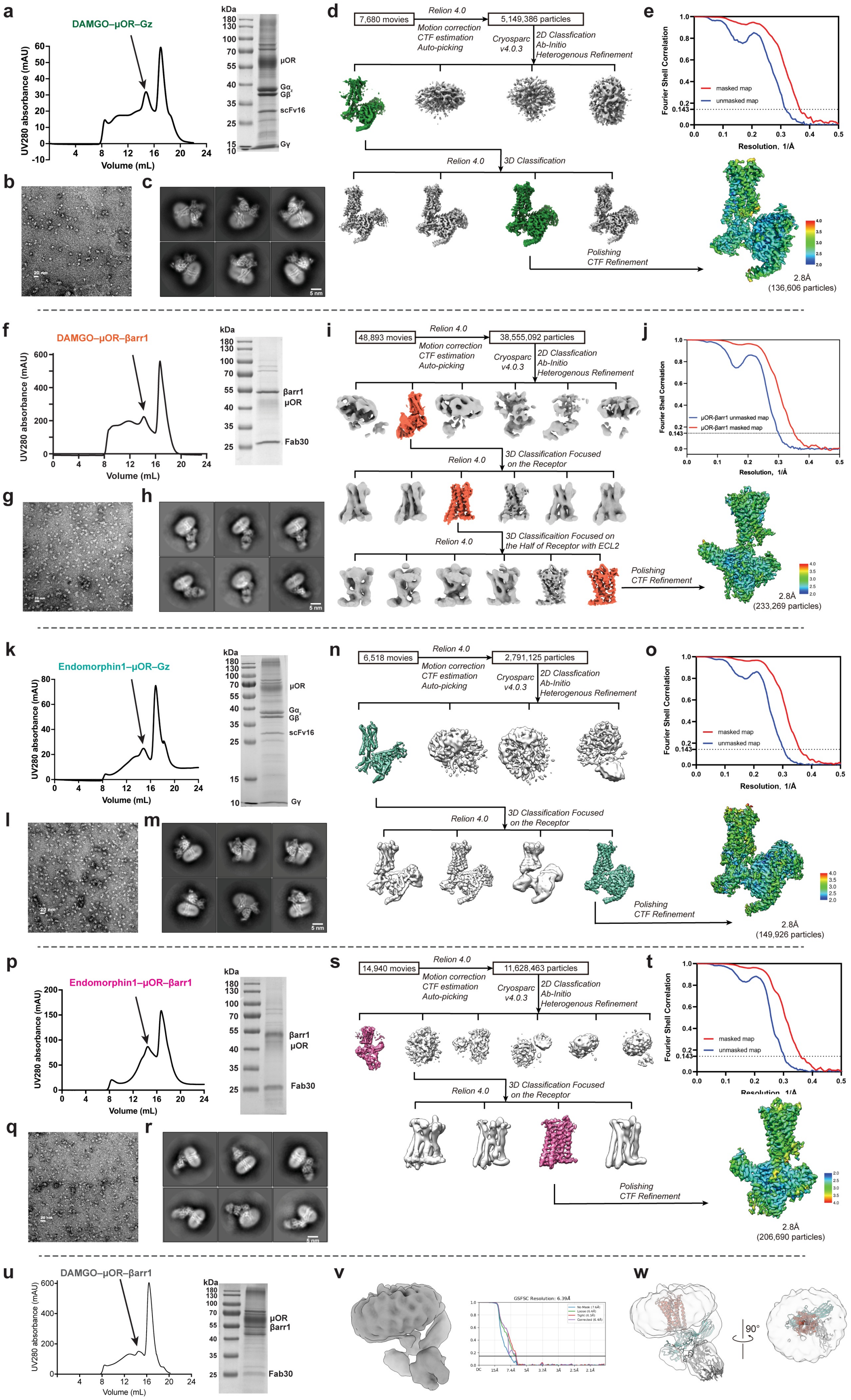

a
DAMGO–μOR–Gz
UV280 absorbance (mAU)
Volume (mL)
kDa
μOR
Gαz
Gβ
scFv16
Gγ
b
c
d
7,680 movies
Relion 4.0
5,149,386 particles
Motion correction
CTF estimation
Auto-picking
Cryosparc v4.0.3
2D Classfication
Ab-Initio
Heterogenous Refinement
3D Classification
Polishing
CTF Refinement
2.8Å
(136,606 particles)
e
Fourier Shell Correlation
masked map
unmasked map
Resolution, 1/Å
f
DAMGO–μOR–βarr1
βarr1
μOR
Fab30
g
h
i
48,893 movies
38,555,092 particles
3D Classification Focused on the Receptor
3D Classification Focused on the Half of Receptor with ECL2
2.8Å
(233,269 particles)
j
μOR-βarr1 unmasked map
μOR-βarr1 masked map
k
Endomorphin1–μOR–Gz
l
m
n
6,518 movies
2,791,125 particles
2.8Å
(149,926 particles)
o
p
Endomorphin1–μOR–βarr1
q
r
s
14,940 movies
11,628,463 particles
2.8Å
(206,690 particles)
t
u
DAMGO–μOR–βarr1
v
GSFSC Resolution: 6.39Å
w
90°

Fig. S1. Preparation and structure determination of DAMGO/endomorphin-1–μOR–G_z–scFv16 complexes and DAMGO/endomorphin-1–μOR–βarr1–Fab30 complexes.

a-e Preparation and structure determination of DAMGO–μOR–G_z–scFv16. Size exclusion chromatography (SEC) profile and sodium dodecyl sulfate polyacrylamide gel electrophoresis (SDS-PAGE) (**a**), negative-stain electron microscopy (EM) (**b**) and 2D class averages (scale bar: 5 nm) of DAMGO–μOR–G_z–scFv16 complex (**c**), flow chart of cryo-EM data processing (details are given in the Method) (**d**), and fourier shell correlation (FSC) curves of the final refined DAMGO–μOR–G_z–scFv16 (**e**).

f-j Preparation and structure determination of DAMGO–μOR (V2R C-tail)–βarr1–Fab30. SEC profile and SDS-PAGE (**f**), negative-stain EM (**g**), 2D class averages (scale bar: 5 nm) of DAMGO–μOR–βarr1–Fab30 complex (**h**), flow chart of cryo-EM data processing (details are given in the Method) (**i**), and FSC curves of the final refined DAMGO–μOR–βarr1–Fab30 complex (**j**).

k-o Preparation and structure determination of endomorphin-1–μOR–G_z–scFv16. SEC profile and SDS-PAGE (**k**), negative-stain EM (**l**) and 2D class averages (scale bar: 5 nm) of endomorphin-1–μOR–G_z–scFv16 complex (**m**), flow chart of cryo-EM data processing (details are given in the Method) (**n**), and FSC curves of the final refined endomorphin-1–μOR–G_z–scFv16 (**o**).

p-t Preparation and structure determination of endomorphin-1–μOR (V2R C-tail)–βarr1–Fab30. SEC profile and SDS-PAGE (**p**), negative-stain EM (**q**), 2D class averages (scale bar: 5 nm) of endomorphin-1–μOR–βarr1–Fab30 complex (**r**), flow chart of cryo-EM data processing (details are given in the Method) (**s**), and FSC curves of the final refined endomorphin-1–μOR–βarr1–Fab30 (**t**).

u SEC profile and SDS-PAGE of DAMGO–μOR^{WT}–βarr1–Fab30 complex. **v** The density map and FSC curves of the final refined DAMGO–μOR^{WT}–βarr1–Fab30 complex. **w** The alignment of DAMGO–μOR^{WT}–βarr1–Fab30 density map and DAMGO–μOR^{V2R}–βarr1–Fab30 atomic model.
