# Supplementary material for: The molecular basis of μ-opioid receptor signaling plasticity
Source: Cell Res. 2025 Nov 7;35(12):1021–36. doi: 10.1038/s41422-025-01191-8 (PMC12689640; doi:10.1038/s41422-025-01191-8)
Supplement: Supplementary file 2 — Supplementary information, Figure S2 [file 41422_2025_1191_MOESM2_ESM.pdf]

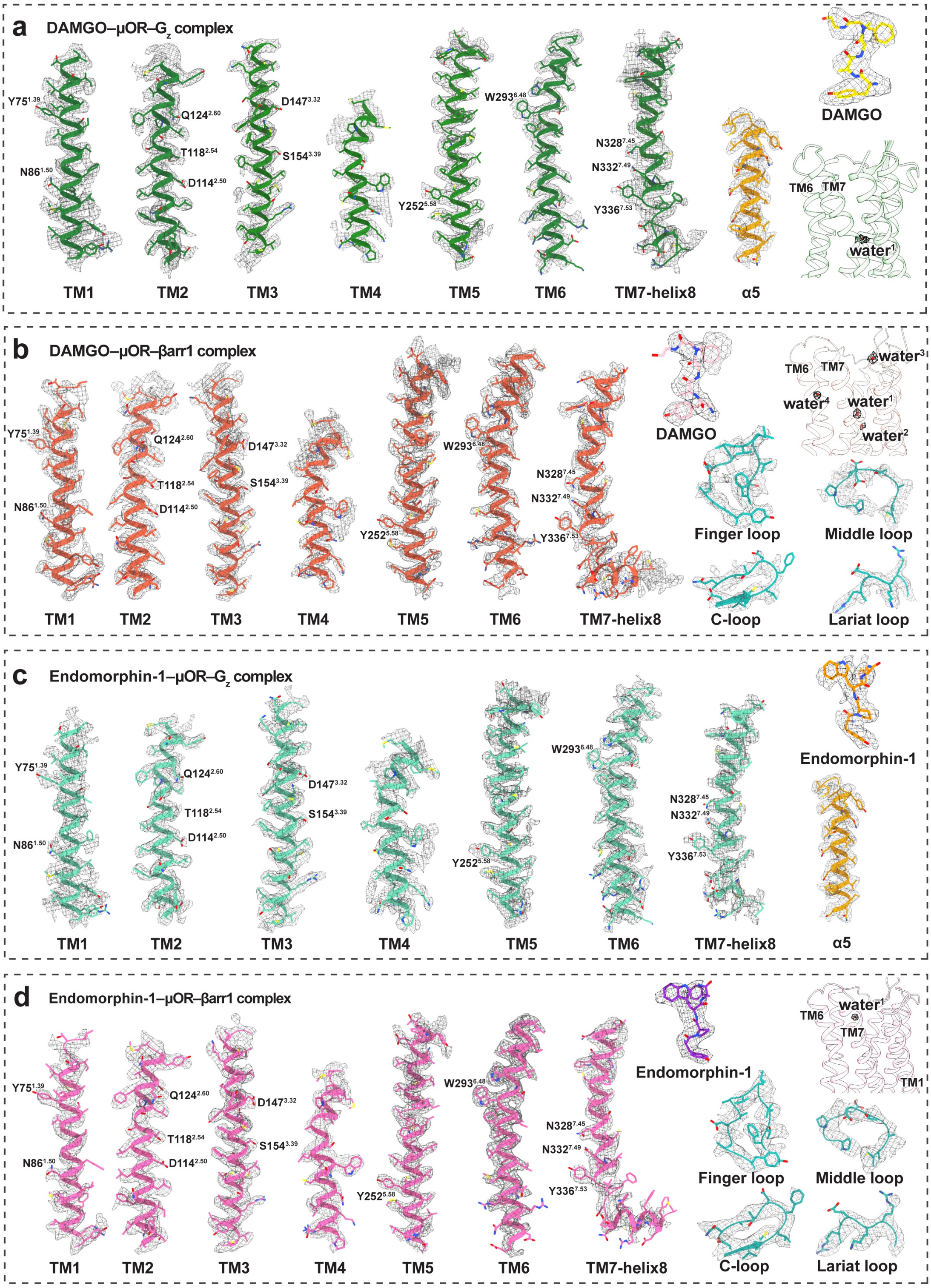

Fig. S2. Cryo-EM density maps and models. **a** Cryo-EM density maps and models are shown for DAMGO, all seven transmembrane helices and helix8 of μOR, the α5 helix of Gα$_z$, and water molecule in the DAMGO–μOR–G$_z$–scFv16 complex (gold, DAMGO; forest green, μOR; orange, α5 helix; red, water). **b** Cryo-EM density maps and models are shown for DAMGO, all seven transmembrane helices and helix8 of μOR, water molecules, and the finger loop, C-loop, middle loop, and lariat loop of βarr1 in the DAMGO–μOR–βarr1–Fab30 complex (light pink, DAMGO; tomato, μOR; light sea green, βarr1; red, water). **c** Cryo-EM density maps and models are shown

for endomorphin-1, all seven transmembrane helices and helix8 of μOR, and the α5 helix of $G\alpha_z$ in the endomorphin-1–μOR–G_z–scFv16 complex (dark orange, endomorphin-1; medium aquamarine, μOR; orange, α5 helix). **d** Cryo-EM density maps and models are shown for endomorphin-1, all seven transmembrane helices and helix8 of μOR, water molecule, and the finger loop, C-loop, middle loop and lariat loop of βarr1 in the endomorphin-1–μOR–βarr1–Fab30 complex (dark orchid, endomorphin-1; hot pink, μOR; light sea green, βarr1; red, water).
