# Supplementary material for: The molecular basis of μ-opioid receptor signaling plasticity
Source: Cell Res. 2025 Nov 7;35(12):1021–36. doi: 10.1038/s41422-025-01191-8 (PMC12689640; doi:10.1038/s41422-025-01191-8)
Supplement: Supplementary file 3 — Supplementary information, Figure S3 [file 41422_2025_1191_MOESM3_ESM.pdf]

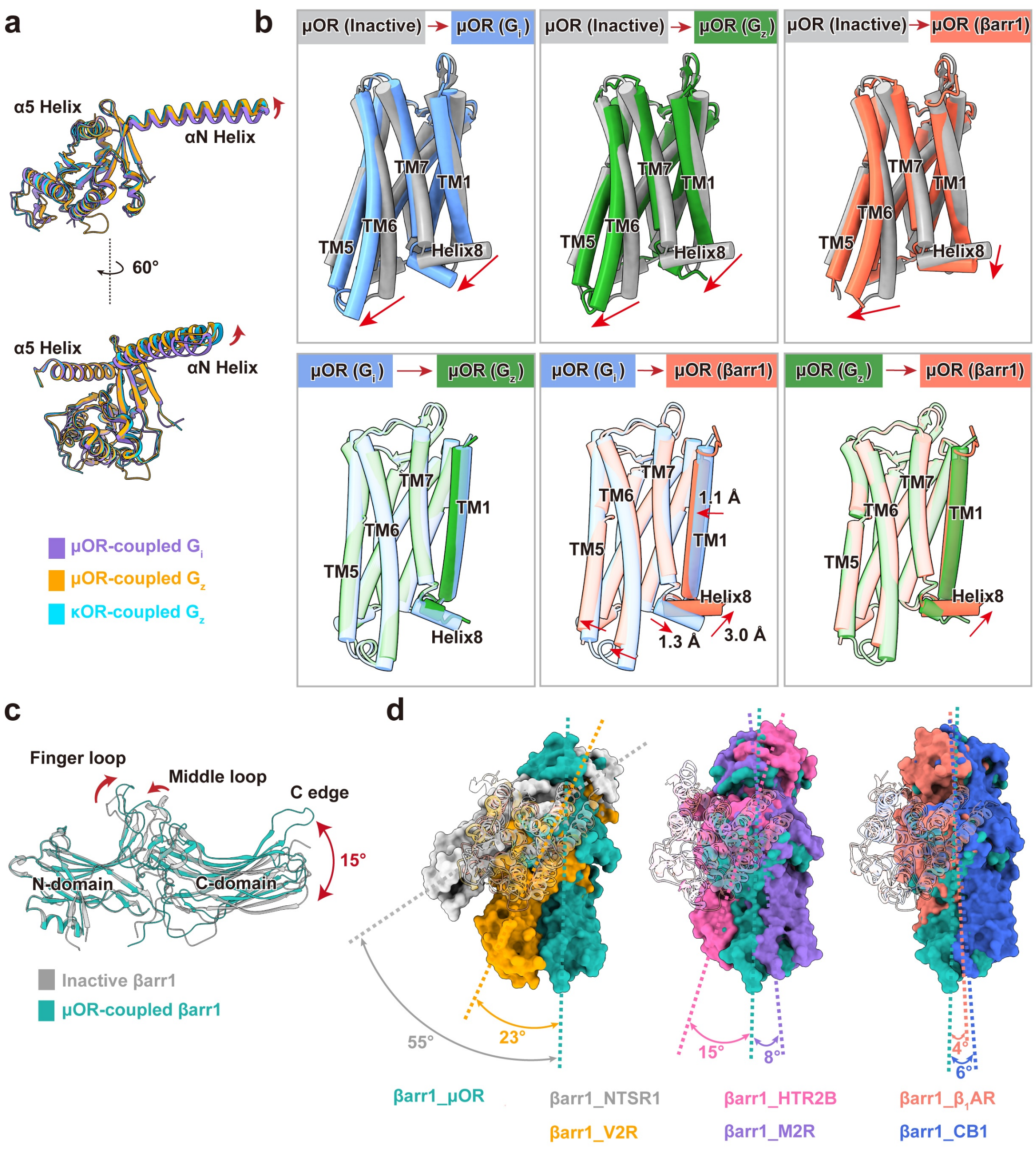

Fig. S3. Structural comparisons of the G_i/G_z/βarr1-coupled μOR complexes. **a** Superposition of the μOR-bounded G_z (orange) and G_i (medium purple; PDB: 8efq), and the κOR-bounded Gz (deep sky blue; PDB: 8dzs). **b** Superposition of the G_i/G_z/βarr1-coupled μOR with the inactive μOR (grey; PDB: 4dkl) and superposition of the G_i/G_z/βarr1-coupled μOR in pairs (cornflower blue, Gi-coupled μOR, PDB: 8efq; forest green, G_z-coupled μOR; tomato, βarr1-coupled μOR). **c** The βarr1 (light sea green) in the μOR–βarr1 complex is superimposed onto the inactive state (grey; PDB: 1g4m). Movements of the central crest loops are indicated by red arrows. The C-lobe is tilted by 15° relative to N-lobe upon activation. **d** Overlay of the μOR–βarr1 (light sea green) structure with NTSR1–βarr1 (light gray; PDB: 6up7), V2R–βarr1 (orange; PDB: 7r0c), HTR2B–βarr1 (hot pink; PDB: 7srs), M2R–βarr1 (medium purple; PDB: 6u1n), $β_1$AR–βarr1 (salmon; PDB: 6tko) and CB1R–βarr1 (royal blue; PDB: 8wu1) structures on the basis of the receptor alignment, viewed from the extracellular. The differences in the orientation of βarr1 in the complexes are given by the angle of rotation.
