# Supplementary material for: The molecular basis of μ-opioid receptor signaling plasticity
Source: Cell Res. 2025 Nov 7;35(12):1021–36. doi: 10.1038/s41422-025-01191-8 (PMC12689640; doi:10.1038/s41422-025-01191-8)
Supplement: Supplementary file 4 — Supplementary information, Figure S4 [file 41422_2025_1191_MOESM4_ESM.pdf]

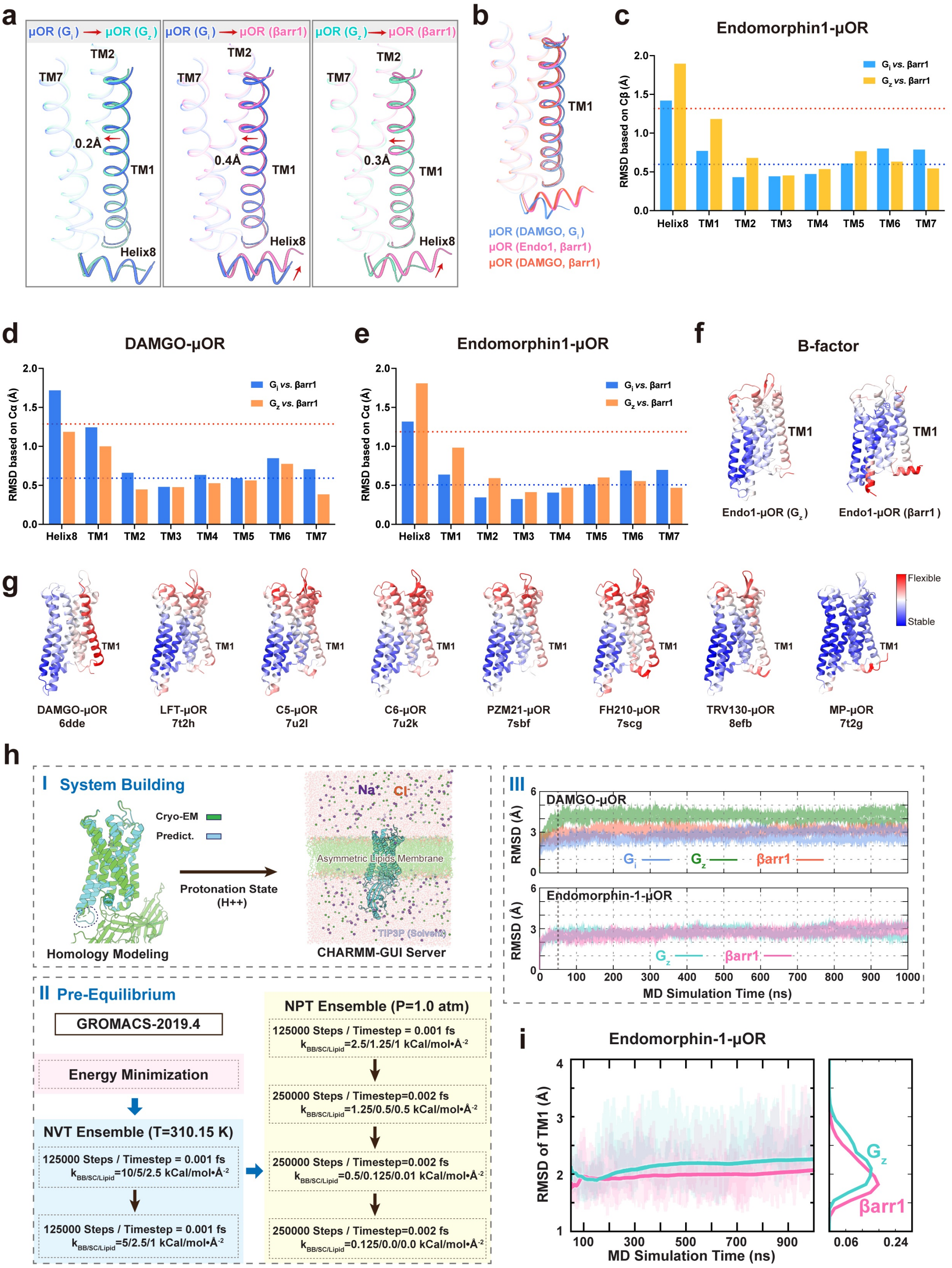

Fig. S4. TM1 dynamics in various adaptor coupling of μOR. a Superposition of βarr1-coupled μOR (hot pink), G_Z-coupled μOR (medium aquamarine) and G_i-coupled μOR (royal blue; PDB: 8f7r) activated by endormorphin-1 to highlight the changes in TM1 and helix8 in pairs. TM1 and helix8 movements indicated by red arrows. **b** Superposition of βarr1-coupled μOR (hot pink) activated by endormorphin-1 (Endo1), βarr1-coupled μOR (tomato) and G_i-coupled μOR (cornflower blue) activated by DAMGO. **c-e** Statistics of the TMs 1-7 and helix8 RMSD of μOR in various adaptor coupling structures, including endomorphin-1-activated μOR based on Cβ (**c**),

DAMGO-activated μOR based on Cα (**d**), and endomorphin-1-activated μOR based on Cα (**e**). **f** B-factor for G_z- and βarr1-coupled μOR both activated by endomorphin-1 (Endo1). **g** B-factor for G_i-coupled μOR activated by various agonists. **h** Schematic diagram of the MD analysis process, including system building, pre-equilibrium simulation, and equilibrium analysis by the RMSD of the complex. **i** MD analysis of the dynamics of TM1 in βarr1-coupled μOR (hot pink), G_z-coupled μOR (medium aquamarine) complexes activated by endomorphin-1.
