# Supplementary material for: The molecular basis of μ-opioid receptor signaling plasticity
Source: Cell Res. 2025 Nov 7;35(12):1021–36. doi: 10.1038/s41422-025-01191-8 (PMC12689640; doi:10.1038/s41422-025-01191-8)
Supplement: Supplementary file 5 — Supplementary information, Figure S5 [file 41422_2025_1191_MOESM5_ESM.pdf]

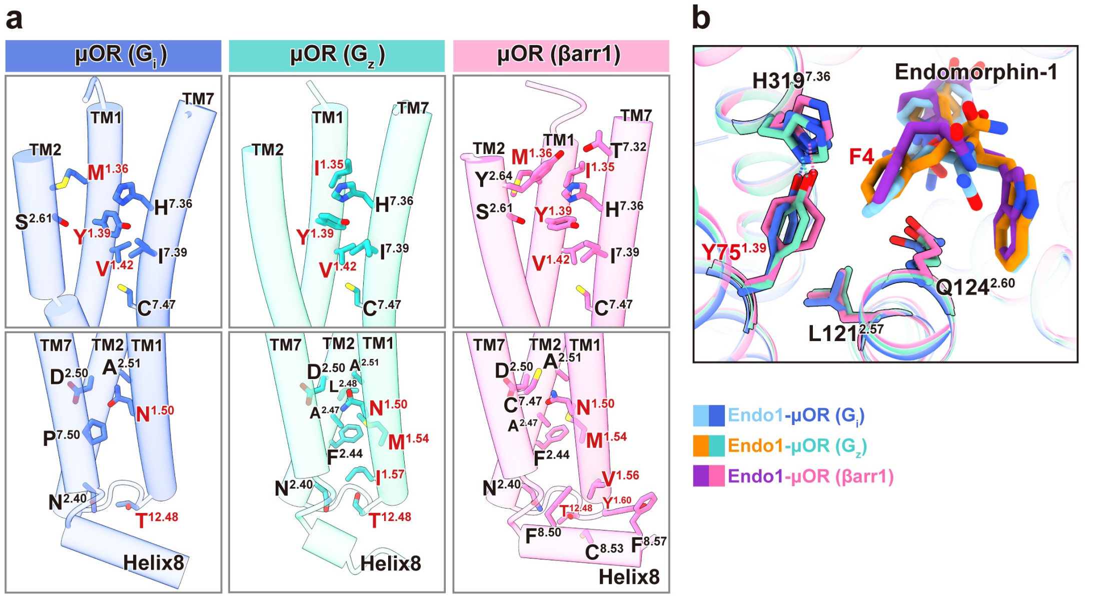

Fig. S5. TM1 regulates μOR downstream signaling selectivity. a Presentation of residues involved in endomorphin-1-activated G_i-, G_z- and βarr1-bound μOR interaction between TM1 and TMs 2/7-helix8. **b** Close-up view of structural differences of the endormorphin-1 and key residues on TM1-fusion pocket.
