# Supplementary material for: The molecular basis of μ-opioid receptor signaling plasticity
Source: Cell Res. 2025 Nov 7;35(12):1021–36. doi: 10.1038/s41422-025-01191-8 (PMC12689640; doi:10.1038/s41422-025-01191-8)
Supplement: Supplementary file 6 — Supplementary information, Figure S6 [file 41422_2025_1191_MOESM6_ESM.pdf]

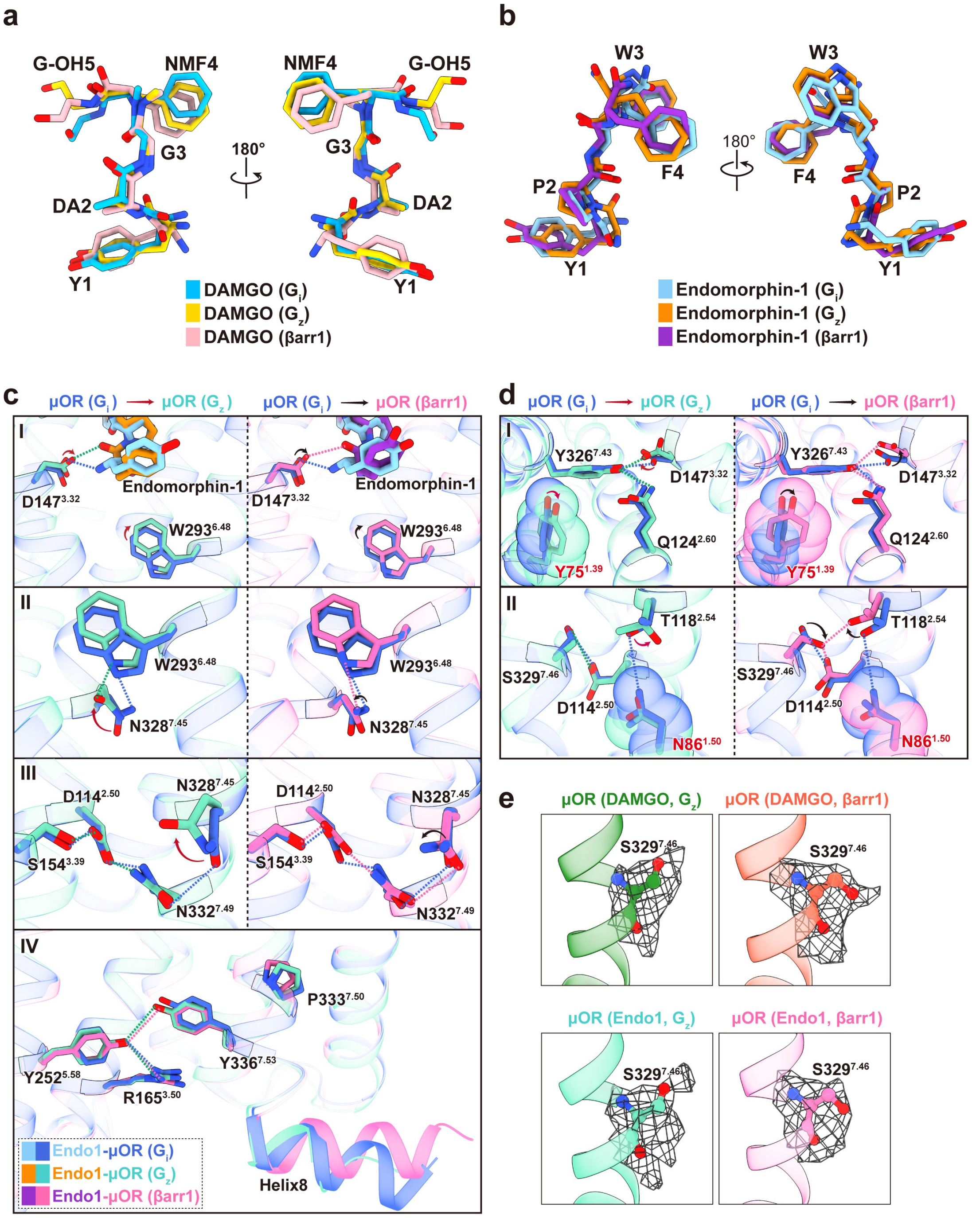

Fig. S6. Comparisons of endomorphin-1 activated μOR in different states. a, b Superposition of DAMGO (**a**) and endomorphin-1 (**b**) in μOR–βarr1, μOR–G_z, and μOR–G_i complexes. **c, d** Close-up views of residues with conformation changes from G_i-bound state to G_z-coupled μOR and from G_i-bound state to βarr1-coupled state. Movements of residues from G_i-bound state to G_z-coupled state are indicated by red arrows, and movements from the G_i-bound state to βarr1-coupled state are indicated by black arrows. **e** Cryo-EM density maps and models of $S329^{7.46}$ in G_z- and βarr1-coupled μOR activated by DAMGO or endomorphin-1 (Endo1).
