# Supplementary material for: The molecular basis of μ-opioid receptor signaling plasticity
Source: Cell Res. 2025 Nov 7;35(12):1021–36. doi: 10.1038/s41422-025-01191-8 (PMC12689640; doi:10.1038/s41422-025-01191-8)
Supplement: Supplementary file 7 — Supplementary information, Figure S7 [file 41422_2025_1191_MOESM7_ESM.pdf]

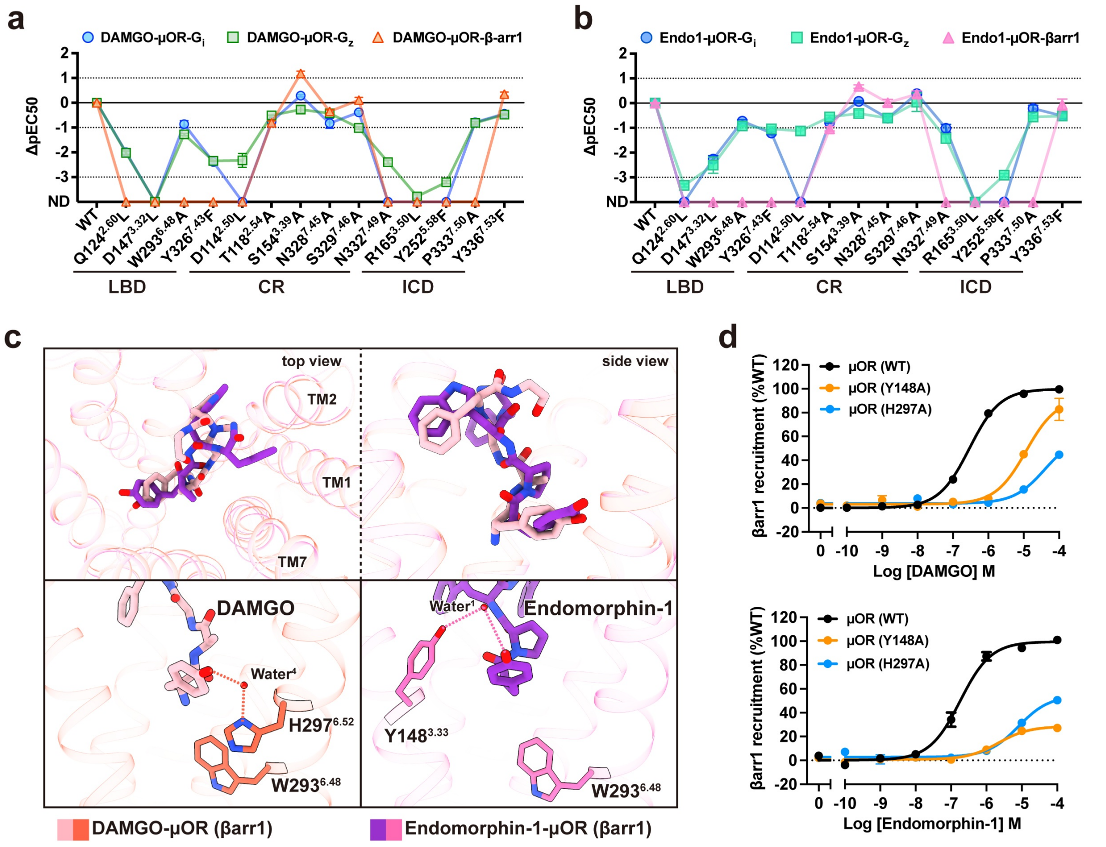

Fig. S7. Differences of DAMGO and endomorphin-1 in activating μOR. a, b Mutagenesis analysis of the key residues in βarr1-, G_z- and G_i-bound μOR activated by DAMGO (**a**) and endomorphin-1 (Endo1) (**b**) using BRET assay. Data represent mean ± SEM of n ≥ 3 biological replicates. **c** Close-up views of residues conformation differences between DAMGO and endomorphin-1 bound to βarr1-coupled μOR during μOR activation. **d** Mutagenesis analysis of $Y148^{3.33}A$ and $H297^{6.52}A$ in the βarr1 recruitment of μOR using BRET assay. Data represent mean ± SEM of n ≥ 3 biological replicates.
