# Supplementary material for: The molecular basis of μ-opioid receptor signaling plasticity
Source: Cell Res. 2025 Nov 7;35(12):1021–36. doi: 10.1038/s41422-025-01191-8 (PMC12689640; doi:10.1038/s41422-025-01191-8)
Supplement: Supplementary file 8 — Supplementary information, Table S1 [file 41422_2025_1191_MOESM8_ESM.pdf]

Table S1. Cryo-EM data collection, model refinement and validation statistics.

	μOR–G_z Complex		μOR–βarr1 Complex	
	DAMGO	Endomorphin-1	DAMGO	Endomorphin-1
**Data collection and processing**				
Magnification	130,000	130,000	130,000	130,000
Voltage (kV)	300	300	300	300
Electron exposure ($e^-/Å^2$)	52	52	52	52
Defocus range (μm)	-0.6 ~ -2.0	-0.6 ~ -2.0	-0.6 ~ -2.0	-0.6 ~ -2.0
Pixel size (Å)	0.93	0.93	0.93	0.93
Symmetry imposed	C1	C1	C1	C1
Initial particle projections (no.)	5,149,386	2,791,125	38,555,092	11,628,463
Final particle projections (no.)	136,606	149,926	233,269	206,690
Map resolution (Å)	2.8	2.8	2.8	2.8
FSC threshold	0.143	0.143	0.143	0.143
Map resolution range (Å)	2.0-4.0	2.0-4.0	2.0-4.0	2.0-4.0
**Refinement**				
Initial model used	6dde	6dde	6dde, 6u1n	6dde, 6u1n
Model resolution (Å)	3.2	3.1	3.1	3.3
FSC threshold	0.5	0.5	0.5	0.5
Model resolution range (Å)	2.0-4.0	2.0-4.0	2.0-4.0	2.0-4.0
Map sharpening B factor ($Å^2$)	-30	-83.94	-63.91	-50
Model composition				
Non-hydrogen atoms	7091	7135	7639	7623
Protein residues	895	901	1021	1023
B factors ($Å^2$)				
Protein	57.85	42.77	45.59	101.05
Ligand	66.41	55.92	27.15	117.98
Water	55.98	36.08	28.01	107.82
R.m.s. deviations				
Bond lengths (Å)	0.003	0.003	0.004	0.002
Bond angles (°)	0.642	0.581	0.711	0.571
Validation				
MolProbity score	1.36	1.42	1.81	1.60
Clashscore	5.50	7.63	9.44	6.76
Rotamer outliers (%)	0.00	0.00	0.00	0.00
Ramachandran plot				
Favored (%)	97.73	97.98	95.56	96.47
Allowed (%)	2.27	2.02	4.44	3.53
Disallowed (%)	0.00	0.00	0.00	0.00
