# Supplementary material for: The molecular basis of μ-opioid receptor signaling plasticity
Source: Cell Res. 2025 Nov 7;35(12):1021–36. doi: 10.1038/s41422-025-01191-8 (PMC12689640; doi:10.1038/s41422-025-01191-8)
Supplement: Supplementary file 9 — Supplementary information, Table S2 [file 41422_2025_1191_MOESM9_ESM.pdf]

Table S2. Cell-surface expression of wild-type (WT) μOR and mutants by ELISA assay.

	Expression[a] (%WT, G protein)	Expression[a] (%WT, βarr1)
**WT**	100	100
**Y75$^{1.39}$A**	105 ± 5	79 ± 12
**Y75$^{1.39}$L**	119 ± 4	108 ± 14
**Y75$^{1.39}$F**	125 ± 4*	93 ± 9
**Y75$^{1.39}$N**	99 ± 6	171 ± 3****
**Y75$^{1.39}$W**	71 ± 3**	85 ± 7
**V78$^{1.42}$A**	110 ± 3	92 ± 8
**V78$^{1.42}$L**	127 ± 6*	104 ± 21
**G82$^{1.46}$A**	84 ± 2	84 ± 6
**N86$^{1.50}$A**	64 ± 6***	56 ± 4
**V89$^{1.53}$A**	65 ± 6***	137 ± 17
**V89$^{1.53}$L**	66 ± 4***	131 ± 10
**I93$^{1.57}$F**	40 ± 3****	27 ± 4***
**T97$^{12.48}$A**	116 ± 7	116 ± 18
**T97$^{12.48}$Y**	93 ± 5	126 ± 25
**D114$^{2.50}$L**	39 ± 2****	31 ± 3***
**T118$^{2.54}$A**	128 ± 7 *	78 ± 9
**Q124$^{2.60}$L**	128 ± 4**	104 ± 7
**D147$^{3.32}$L**	62 ± 11****	65 ± 4
**Y148$^{3.33}$A**	/	159 ± 13**
**S154$^{3.39}$A**	84 ± 5	54 ± 8
**R165$^{3.50}$L**	155 ± 8****	170 ± 13****
**Y252$^{5.58}$F**	115 ± 5	138 ± 11
**W293$^{6.48}$A**	99 ± 6	87 ± 4
**H297$^{6.52}$A**	/	49 ± 9**
**Y326$^{7.43}$F**	71 ± 6**	62 ± 7
**N328$^{7.45}$A**	96 ± 6	106 ± 12
**S329$^{7.46}$A**	113 ± 8	97 ± 4
**N332$^{7.49}$A**	81 ± 6	67 ± 8
**P333$^{7.50}$A**	70 ± 4**	87 ± 6
**Y336$^{7.53}$F**	95 ± 7	95 ± 18
**T97$^{12.48}$A/S329$^{7.49}$A**	135 ± 3***	143 ± 3*

[a] Data are shown as mean ± SEM from at least three independent experiments performed in technical triplicate. The significance was determined by one-way ANOVA followed by Dunnett's multiple comparisons test compared with the WT. $^{*}P < 0.05$, $^{**}P < 0.01$, $^{***}P < 0.001$ and $^{****}P < 0.0001$ were considered as statistically significant.
