# Supplementary material for: The molecular basis of μ-opioid receptor signaling plasticity
Source: Cell Res. 2025 Nov 7;35(12):1021–36. doi: 10.1038/s41422-025-01191-8 (PMC12689640; doi:10.1038/s41422-025-01191-8)
Supplement: Supplementary file 10 — Supplementary information, Table S3 [file 41422_2025_1191_MOESM10_ESM.pdf]

**Table S3. Effects of DAMGO on Gα$_i$–Gβγ dissociation, Gα$_z$–Gβγ dissociation and β-arrestin-1 recruitment in WT and mutants of μOR by BRET1 assay.**

| | Gα$_i$–Gβγ dissociation | | Gα$_z$–Gβγ dissociation | | β-arrestin-1 recruitment | |
|---|---|---|---|---|---|---|
| | pEC50 ± SEM[a] | Span±SEM[a,b] (%WT) | pEC50 ± SEM[a] | Span±SEM[a,b] (%WT) | pEC50 ± SEM[a] | Span±SEM[a,b] (%WT) |
| **WT** | 8.34±0.03 | 100.00±0.00 | 9.29±0.03 | 100.00±0.00 | 6.41±0.02 | 100.00±0.00 |
| **Y75$^{1.39}$A** | ND[c] | ND[c] | 5.71±0.02$^{****}$ | 234.35±10.62$^{****}$ | ND[c] | ND[c] |
| **Y75$^{1.39}$L** | 5.54±0.06$^{****}$ | 95.74±0.73 | 7.01±0.09$^{****}$ | 144.31±19.33$^{*}$ | ND[c] | ND[c] |
| **Y75$^{1.39}$F** | 6.52±0.21$^{****}$ | 87.14±3.49 | 7.38±0.11$^{****}$ | 134.94±15.91 | 4.88±0.04$^{****}$ | 39.5±1.75$^{****}$ |
| **Y75$^{1.39}$N** | 7.37±0.10$^{****}$ | 92.05±1.88 | 8.31±0.02$^{****}$ | 136.50±6.99 | 5.29±0.11$^{****}$ | 72.07±5.47$^{**}$ |
| **Y75$^{1.39}$W** | 6.21±0.19$^{****}$ | 81.29±3.35 | 7.61±0.23$^{****}$ | 98.84±10.66 | ND[c] | ND[c] |
| **V78$^{1.42}$A** | 7.23±0.09$^{****}$ | 86.21±0.87 | 8.40±0.13$^{****}$ | 131.64±10.72 | 5.16±0.12$^{****}$ | 84.04±5.61 |
| **V78$^{1.42}$L** | 7.84±0.09$^{**}$ | 92.15±4.74 | 8.76±0.15$^{*}$ | 104.95±1.08 | 5.75±0.07$^{**}$ | 95.44±4.14 |
| **G82$^{1.46}$A** | 8.07±0.12 | 69.97±2.40$^{**}$ | 8.96±0.03 | 90.56±9.40 | 6.49±0.07 | 46.92±1.37$^{****}$ |
| **N86$^{1.50}$A** | 7.11±0.10$^{****}$ | 56.25±2.67$^{****}$ | 8.17±0.08$^{****}$ | 127.03±6.64 | ND[c] | ND[c] |
| **V89$^{1.53}$A** | 8.11±0.06 | 104.20±6.34 | 9.17±0.08 | 150.37±6.49$^{**}$ | 6.85±0.12 | 50.52±3.84$^{****}$ |
| **V89$^{1.53}$L** | 7.89±0.20$^{*}$ | 59.84±0.54$^{****}$ | 8.71±0.13$^{**}$ | 120.95±17.17 | ND[c] | ND[c] |
| **I93$^{1.57}$F** | 7.45±0.09$^{****}$ | 56.31±5.08$^{****}$ | 8.32±0.18$^{****}$ | 83.11±9.17 | ND[c] | ND[c] |
| **T97$^{12.48}$A** | 8.49±0.05 | 75.91±0.52$^{*}$ | 9.22±0.14 | 51.31±5.20$^{**}$ | 6.42±0.05 | 82.39±8.35 |
| **T97$^{12.48}$Y** | 7.78±0.07$^{***}$ | 87.77±4.82 | 8.67±0.07$^{**}$ | 117.94±7.10 | 6.26±0.11 | 51.40±6.02$^{****}$ |
| **D114$^{2.50}$L** | ND[c] | ND[c] | 6.96±0.28$^{****}$ | 73.74±3.10 | ND[c] | ND[c] |
| **T118$^{2.54}$A** | 7.57±0.07$^{****}$ | 106.14±3.33 | 8.78±0.01$^{*}$ | 109.48±1.97 | 5.60±0.08$^{****}$ | 99.40±6.20 |
| **Q124$^{2.60}$L** | 6.34±0.08$^{****}$ | 61.17±7.42$^{****}$ | 7.27±0.07$^{****}$ | 158.02±4.73$^{***}$ | ND[c] | ND[c] |
| **D147$^{3.32}$L** | ND[c] | ND[c] | ND[c] | ND[c] | ND[c] | ND[c] |
| **Y148$^{3.33}$A** | / | / | / | / | 4.95±0.08$^{****}$ | 89.57±12.41 |
| **S154$^{3.39}$A** | 8.62±0.04 | 61.90±3.94$^{****}$ | 9.01±0.13 | 60.89±8.46 | 7.59±0.09$^{****}$ | 76.23±2.07$^{*}$ |
| **R165$^{3.50}$L** | ND[c] | ND[c] | 5.51±0.12$^{****}$ | 36.57±2.00$^{***}$ | ND[c] | ND[c] |
| **Y252$^{5.58}$F** | ND[c] | ND[c] | 6.08±0.06$^{****}$ | 214.26±21.04$^{****}$ | ND[c] | ND[c] |
| **W293$^{6.48}$A** | 7.47±0.14$^{****}$ | 68.58±8.19$^{***}$ | 8.01±0.04$^{****}$ | 63.89±8.17 | ND[c] | ND[c] |
| **H297$^{6.52}$A** | / | / | / | / | 4.36±0.16$^{****}$ | 40.54±3.23$^{****}$ |
| **Y326$^{7.43}$F** | 5.95±0.06$^{****}$ | 74.87±8.71$^{**}$ | 6.94±0.03$^{****}$ | 195.14±5.10$^{****}$ | ND[c] | ND[c] |
| **N328$^{7.45}$A** | 7.51±0.21$^{****}$ | 60.72±5.51$^{****}$ | 8.86±0.13$^{*}$ | 113.24±11.17 | 6.06±0.06 | 57.27±4.95$^{****}$ |
| **S329$^{7.46}$A** | 7.95±0.07 | 62.59±1.08$^{****}$ | 8.27±0.05$^{****}$ | 28.46±2.38$^{****}$ | 6.52±0.11 | 81.94±1.26 |
| **N332$^{7.49}$A** | ND[c] | ND[c] | 6.90±0.08$^{****}$ | 110.97±2.91 | ND[c] | ND[c] |
| **P333$^{7.50}$A** | 7.55±0.10$^{****}$ | 30.20±5.18$^{****}$ | 8.48±0.05$^{****}$ | 111.51±6.41 | ND[c] | ND[c] |
| **Y336$^{7.53}$F** | 7.89±0.05$^{*}$ | 120.29±7.55 | 8.81±0.03 | 135.31±7.12 | 6.76±0.08 | 72.38±2.60$^{***}$ |
| **T97$^{12.48}$A/S329$^{7.49}$A** | 8.46±0.03 | 64.91±5.79$^{***}$ | ND[c] | ND[c] | 6.68±0.01 | 55.34±6.53$^{****}$ |

[a] Data were analyzed using a three-parameter logistic equation to determine potency (pEC50) and efficacy (span). Data are shown as mean ± SEM from at least three independent experiments performed in technical triplicate. $^{*}P < 0.05$, $^{**}P < 0.01$, $^{***}P < 0.001$ and $^{****}P < 0.0001$ were determined by one-way ANOVA followed by Dunnett's multiple comparisons test, compared with the response of the WT.

[b] The span is defined as the window between the maximal response ($E_{max}$) and the vehicle (no DAMGO). Data were normalized to wild-type which was set to 100%.

[c] ND (not detectable) refers to data where a robust concentration response curve could not be established within the concentration range tested or the span < 20%.
