# Supplementary material for: The molecular basis of μ-opioid receptor signaling plasticity
Source: Cell Res. 2025 Nov 7;35(12):1021–36. doi: 10.1038/s41422-025-01191-8 (PMC12689640; doi:10.1038/s41422-025-01191-8)
Supplement: Supplementary file 11 — Supplementary information, Table S4 [file 41422_2025_1191_MOESM11_ESM.pdf]

Table S4. Effects of endomorphin-1 on Gα$_i$-Gβγ dissociation, Gα$_z$-Gβγ dissociation and β-arrestin-1 recruitment in WT and mutants of μOR by BRET1 assay.

	Gα$_i$-Gβγ dissociation		Gα$_z$-Gβγ dissociation		β-arrestin-1 recruitment	
	**pEC50 ± SEM^{a}**	**Span±SEM^{a,b} (%WT)**	**pEC50 ± SEM^{a}**	**Span±SEM^{a,b} (%WT)**	**pEC50 ± SEM^{a}**	**Span±SEM^{a,b} (%WT)**
**WT**	8.58±0.03	100.00±0.00	9.39±0.03	100.00±0.00	6.72±0.02	100.00±0.00
**Y75$^{1.39}$A**	5.65±0.17****	103.57±3.99	6.86±0.20****	149.78±10.56**	ND^{c}	ND^{c}
**Y75$^{1.39}$L**	6.83±0.18****	96.18±4.81	8.03±0.10****	131.04±7.10	5.34±0.08****	28.89±2.58****
**Y75$^{1.39}$F**	8.26±0.11	77.09±7.74*	9.16±0.12	108.12±6.20	6.37±0.07*	98.00±5.59
**Y75$^{1.39}$N**	7.76±0.17****	90.31±6.37	8.28±0.05****	181.33±11.86****	5.55±0.04****	78.22±2.61**
**Y75$^{1.39}$W**	7.33±0.05****	80.67±2.72	8.11±0.06****	108.29±5.08	5.47±0.04****	37.03±0.99****
**V78$^{1.42}$A**	7.14±0.20****	81.40±3.61	7.96±0.02****	118.78±7.12	5.49±0.13****	37.47±3.05****
**V78$^{1.42}$L**	8.41±0.09	94.61±2.28	9.21±0.08	107.90±10.59	6.18±0.10****	58.30±6.79****
**G82$^{1.46}$A**	8.47±0.16	83.56±3.58	9.27±0.07	90.67±8.00	6.78±0.11	44.99±7.80****
**N86$^{1.50}$A**	7.17±0.17****	45.05±8.46****	8.08±0.07****	84.70±12.72	ND^{c}	ND^{c}
**V89$^{1.53}$A**	8.34±0.14	80.54±1.96	9.20±0.12	81.26±5.80	7.15±0.05**	42.73±3.90****
**V89$^{1.53}$L**	8.40±0.09	69.59±4.18***	8.84±0.04*	87.27±5.59	ND^{c}	ND^{c}
**I93$^{1.57}$F**	7.65±0.06****	58.23±2.72****	8.90±0.08*	84.08±1.86	ND^{c}	ND^{c}
**T97$^{12.48}$A**	8.87±0.05	84.09±6.94	9.09±0.25	68.14±6.12	6.92±0.06	99.02±3.14
**T97$^{12.48}$Y**	7.93±0.02***	77.49±5.52*	9.22±0.11	96.99±4.96	6.54±0.09	49.30±5.21****
**D114$^{2.50}$L**	ND^{c}	ND^{c}	8.27±0.17****	65.68±1.53	ND^{c}	ND^{c}
**T118$^{2.54}$A**	7.79±0.03****	96.74±4.84	8.84±0.06**	122.89±2.94	5.69±0.09****	69.85±7.21****
**Q124$^{2.60}$L**	ND^{c}	ND^{c}	6.07±0.06****	85.30±4.37	ND^{c}	ND^{c}
**D147$^{3.32}$L**	6.32±0.14****	58.54±3.84****	6.89±0.33****	85.87±5.34	ND^{c}	ND^{c}
**Y148$^{3.33}$A**	/	/	/	/	5.71±0.15****	27.94±1.81****
**S154$^{3.39}$A**	8.66±0.03	64.49±3.97****	8.98±0.12	45.37±1.31***	7.40±0.07****	54.59±2.43****
**R165$^{3.50}$L**	ND^{c}	ND^{c}	ND^{c}	ND^{c}	ND^{c}	ND^{c}
**Y252$^{5.58}$F**	ND^{c}	ND^{c}	6.48±0.06****	109.92±12.48	ND^{c}	ND^{c}
**W293$^{6.48}$A**	7.85±0.07****	35.68±2.08****	8.47±0.02****	62.24±4.23*	ND^{c}	ND^{c}
**H297$^{6.52}$A**	/	/	/	/	5.09±0.09****	52.03±2.61****
**Y326$^{7.43}$F**	7.38±0.07****	68.95±3.80****	8.35±0.08****	103.43±0.94	ND^{c}	ND^{c}
**N328$^{7.45}$A**	7.97±0.09**	53.98±3.32****	8.79±0.12**	100.83±8.16	6.74±0.13	34.67±2.73****
**S329$^{7.46}$A**	8.97±0.06	55.99±4.00****	9.43±0.37	43.48±5.08***	7.08±0.14*	77.27±1.35**
**N332$^{7.49}$A**	7.57±0.16****	25.91±3.86****	7.96±0.18****	87.70±8.81	ND^{c}	ND^{c}
**P333$^{7.50}$A**	8.37±0.13	38.27±2.87****	8.83±0.06**	98.96±8.44	ND^{c}	ND^{c}
**Y336$^{7.53}$F**	8.07±0.12*	73.59±4.07**	8.88±0.06*	116.05±3.73	6.67±0.22	48.60±3.17****
**T97$^{12.48}$A/S329$^{7.49}$A**	9.23±0.13***	61.07±7.00****	ND^{c}	ND^{c}	7.08±0.13*	61.00±3.07****

[a] Data were analyzed using a three-parameter logistic equation to determine potency (pEC50) and efficacy (span). Data are shown as mean ± SEM from at least three independent experiments performed in technical triplicate. $^{*}P < 0.05$, $^{**}P < 0.01$, $^{***}P < 0.001$ and $^{****}P < 0.0001$ were determined by one-way ANOVA followed by Dunnett's multiple comparisons test, compared with the response of the WT.

[b] The span is defined as the window between the maximal response (E_{max}) and the vehicle (no endomorphin-1). Data were normalized to wild-type which was set to 100%.

[c] ND (not detectable) refers to data where a robust concentration response curve could not be established within the concentration range tested or the span < 20%.
