# Supplementary material for: The molecular basis of μ-opioid receptor signaling plasticity
Source: Cell Res. 2025 Nov 7;35(12):1021–36. doi: 10.1038/s41422-025-01191-8 (PMC12689640; doi:10.1038/s41422-025-01191-8)
Supplement: Supplementary file 12 — Supplementary information, Table S5 [file 41422_2025_1191_MOESM12_ESM.pdf]

Table S5. Effects of peptides on $G\alpha_i$-$G\beta\gamma$ dissociation, $G\alpha_z$-$G\beta\gamma$ dissociation and β-arrestin-1 recruitment in WT μOR by BRET1 assay.

	$G\alpha_i$-$G\beta\gamma$ dissociation		$G\alpha_z$-$G\beta\gamma$ dissociation		β-arrestin-1 recruitment	
	pEC50 ± SEM[a]	Span±SEM[a,b] (%WT)	pEC50 ± SEM[a]	Span±SEM[a,b] (%WT)	pEC50 ± SEM[a]	Span±SEM[a,b] (%WT)
**Endomorphin-1**	8.49±0.07	100.00±0.00	9.29±0.09	100.00±0.00	6.61±0.04	100.00±0.00
**P1**	6.38±0.18****	89.93±5.79	7.24±0.08****	92.74±2.92	ND[c]	ND[c]
**P2**	7.40±0.10****	95.25±6.29	8.25±0.12****	92.99±3.80	5.67±0.05	53.41±2.68****
**P3**	6.66±0.04****	87.17±4.77	7.33±0.14****	104.46±5.38	ND[c]	ND[c]

[a] Data were analyzed using a three-parameter logistic equation to determine potency (pEC50) and efficacy (span). Data are shown as mean ± SEM from at least three independent experiments performed in technical triplicate. $^{*}P < 0.05$, $^{**}P < 0.01$, $^{***}P < 0.001$ and $^{****}P < 0.0001$ were determined by one-way ANOVA followed by Dunnett's multiple comparisons test, compared with the response of endomorphin-1.

[b] The span is defined as the window between the maximal response (E_{max}) and the vehicle (no peptide). Data were normalized to endomorphin-1 which was set to 100%.

[c] ND (not detectable) refers to data where a robust concentration response curve could not be established within the concentration range tested or the span < 20%.
